# Effectiveness of post-abortion care services to protect women’s fertility in China: A systematic review with meta-analysis

**DOI:** 10.1371/journal.pone.0304221

**Published:** 2024-06-10

**Authors:** Xin Wang, Mengcong Deng, Yujia Zhu, Shangchun Wu, Qunxia Mao, Hongwei Wang

**Affiliations:** 1 National Research Institute for Family Planning, Beijing, China; 2 Graduate School, Peking Union Medical College & Chinese Academy of Medical Science, Beijing, China; 3 China University of Labor Relations, Beijing, China; Delta State University, NIGERIA

## Abstract

**Objective:**

This study aimed to evaluate the effectiveness of post-abortion care services in Chinese women who have undergone induced abortion.

**Methods:**

A systematic literature search was conducted in five databases from January 2011 to June 2023 (PROSPERO registration CRD42023440458). Estimates of intervention effects were represented as relative risk (RR) with 95% confidence intervals (CI). The Grading of Recommendations Assessment, Development and Evaluation (GRADE) was used to evaluate the strength of recommendations.

**Results:**

The meta-analysis of 42 randomized controlled studies involving 70,126 participants indicated that post-abortion care services could significantly increase rate of effective contraceptive use (RR = 2.33, 95%CI = 1.80–3.00, 10 studies, GRADE (Medium)), reduce repeat abortion rate (RR = 0.26, 95%CI = 0.20–0.36, 19 studies, GRADE (High)), increase follow-up visit rate (RR = 1.37, 95%CI = 1.06–1.75, 5 studies, GRADE (Very low)) in one year after abortions, and improve patient satisfaction rate (RR = 1.37, 95%CI = 1.03–1.83, 9 studies, GRADE (High)).

**Conclusion:**

Post-abortion care services could help increase the rate of continuation of post-abortion effective contraceptives, prevent repeat abortions, and promote female fertility. Exploring strategies for better provision of post-abortion services requires more high-quality research.

## Introduction

Induced abortion is the termination of pregnancy by artificial means [1, 2]. Post-abortion care (PAC) services for women who have had abortions have become one of the primary measures to reduce repeat abortions internationally [3]. PAC was introduced in the International Project Assistance Services’ 1991 strategic planning document, then supported by the United States Agency for International Development since 1994 [4]. World Health Organization (WHO) released post-abortion family planning guide in 1997 [5], later updated in 2022 [6]. In September 2011, China Women’s Development Foundation (CWDF), National Research Institute for Family Planning (NRIFP), Chinese Medical Family Planning Association, and People’s Daily Online jointly initiated the Caring for Post-Abortion Women program, representing the official launch of the PAC programme in China. In 2013, the four-phase collaborative research project funded by European Commission (EC) under the Seventh Framework Programme (FP7) on INtegrating Post-Abortion family planning services into existing abortion services in hospital settings in China (INPAC) is being undertaken, and NRIFP was a core member of the project [7].

Post-abortion family planning service guideline published in China provides a standardized guideline for the widespread implementation of PAC services in China [8]. The goal is to recommend immediate post-operative use of effective contraceptive methods according to the patient’s situation and to raise their awareness of health care and voluntarily adhere to contraception on a long-term basis, to avoid repeat unintended pregnancies and reduce the risk of repeat abortion [9]. PAC services emphasize promoting contraceptive knowledge through public education and personalized counselling for women undergoing induced abortion and their male partners, helping them to promptly implement effective contraceptive measures [4].

More than a decade of PAC services’ implementation has generally proven its practicability and effectiveness based on relative program performance evaluation reports and clinical trials [10, 11]. The INPAC group has also previously made a preliminary positive evaluation [12]. A Chinese language meta-analysis, published in 2017, assessed the positive effectiveness of post-abortion family planning services [13]. Owing to changes in demographic characteristics and more relevant trials in recent years, we conducted an updated meta-analysis to comprehensively and thoroughly evaluate the effectiveness of PAC services in China through several straightforward outcome indicators in two packages of interventions. In addition, it is intended to further complement the data support for future health policy making.

## Methods

This study followed the Preferred Reporting Items for Systematic Reviews and Meta-Analyses (PRISMA) guidelines [14], and its protocol was registered in the International Prospective Register of Systematic Reviews (PROSPERO) database: CRD42023440458.

### Eligibility criteria

#### Population

Chinese women who have undergone induced abortions aged 15–49 years were eligible for inclusion. We excluded women with a diagnosis of serious mental illness and other unsuitable medical conditions for inclusion in this study.

#### Intervention

The following two packages of interventions were eligible. The one was normal PAC services (NPS) following updated guidelines. The NPS procedures included public education, personalized consultation, guidance on the immediate implementation of effective contraceptive measures after abortion, and follow-up at 1, 3, 6, and 12 months post-abortion by telephone call or subsequent visit [8, 15]. The content of education and counselling mainly included risks of induced abortion, the importance of promptly using contraceptives post-abortion, suitable contraceptive methods, and helping to address patients’ doubts and concerns, etc. Another intervention was improved PAC services (IPS) which went beyond NPS, including but not limited to improvements in service format, content, and timing. This may include utilizing electronic platforms, social media applications, and emphasizing humanistic care.

#### Comparison

Any comparisons including routine care or no interventions were both considerable. In the case of studies delivering improved services, normal PAC service was set as the comparison group.

#### Outcomes

The primary outcomes were the rate of effective contraceptive use and the rate of repeat abortion. Based on the definition from WHO, effective contraceptives included intrauterine devices (IUD), implants, injectables, sterilization, combined oral contraceptives (COC), combined contraceptive patch and combined contraceptive vaginal ring [16–18].

The secondary outcomes included follow-up rate and patient satisfaction. Patient satisfaction was the cumulative proportion of self-reported service satisfaction, measured through self-developed questionnaires after the participants received PAC services in the hospitals. The rates of effective contraceptive use, repeat abortion, and follow-up were calculated at 1, 3, 6, and 12 months post-abortion, as well as the rate of immediate effective contraceptive use.

#### Study design

Only randomized controlled trials (RCT) in full-text published were eligible for inclusion. Studies whose follow-up time was less than 3 months after abortions; sample size was less than 100 [19]; master’s or doctoral thesis or research report; studies that did not report outcomes of interest; multiple submissions and duplicate publications were excluded.

### Search strategy

English electronic databases (PubMed, EMBASE, Web of Science), WHO ICTRP, and Chinese electronic databases (CNKI and Wanfang) were comprehensively and systematically searched from January 2011 to June 2023. Bibliographies of the retrieved articles were also hand-searched to identify any relevant articles for this review. Search terms and search strategies were in S1 Appendix.

### Data screening and extraction process

Two authors (X.W. and M.D.) were assigned to independently screen the titles and abstracts among the records organized in Endnote X9 to retrieve relevant records. Then, they were also assigned to independently perform the second screening of the full text based on the predefined inclusion criteria, and independently extracted data from included studies using a format prepared in a Microsoft Excel spreadsheet. Detailed information was extracted, including first authors, year of publication, study designs, sample sizes, ages, length of follow-up, follow-up methods, interventions, comparisons and outcomes, etc. Any disagreement was resolved by discussion until consensus was reached or by consulting a third author (Y.Z.). Eligibility for inclusion in the meta-analysis was also determined for each study.

### Risk of bias

We evaluated the quality of the studies based on the Cochrane “risk of bias” assessment tool and using criteria outlined in the Revised Cochrane risk-of-bias tool for Randomized Trials (RoB 2) [20]. Based on the rating obtained from 5 domains, each study was classified as having “Low risk”, “High risk”, and “Some Concerns”. The risk of bias was assessed by two authors independently (X.W. and Q.M.). Any discrepancies were discussed until a consensus was reached (S.W.). A summary figure of the assessed bias of the included studies was created using Review Manager 5.4.

### Publication bias and heterogeneity

Rigorous searches (electronic/database search and manual search) have been used to minimize the risk of bias. Publication bias was assessed by funnel plots, and quantitative analysis was performed by Peter’s method [21]. The trim and fill method was utilized to determine potential publication bias and compute an imputed effect value [22].

According to the Cochrane Handbook criteria, the Higgins I^2^ test measured heterogeneity among studies with its corresponding p-value. I^2^ test statistics values of 0, 25, 50, and 75% were considered no, low, moderate, and high degrees of heterogeneity, respectively. In this study, when I^2^>50%, there was an obvious heterogeneity and the random effect model would be used, otherwise, the fixed effect model would be applied [23, 24].

### Data synthesis

The estimated effectiveness regarding effective contraceptive use, repeat abortion and follow-up during 1 year after induced abortion, and patient satisfaction were expressed as relative risk (RR) and 95% confidence interval (CI). Where studies measured the same outcomes, we included them in a meta-analysis. Sensitivity analyses were performed to explore possible explanations for heterogeneity by leave-one-out influence analysis and excluding high-risk-of-bias trials. The data syntheses were done using R-studio Version 1.1.383(1999 Free Software Foundation, Boston, Massachusetts, MA, USA: Rstudio, PBC).

### Grading the certainty of evidence

The Grading of Recommendations, Assessment, Development, and Evaluations (GRADE) approach has been used to rate the overall certainty of the evidence [25].

## Results

### Quantity of literature available

**Fig 1:**
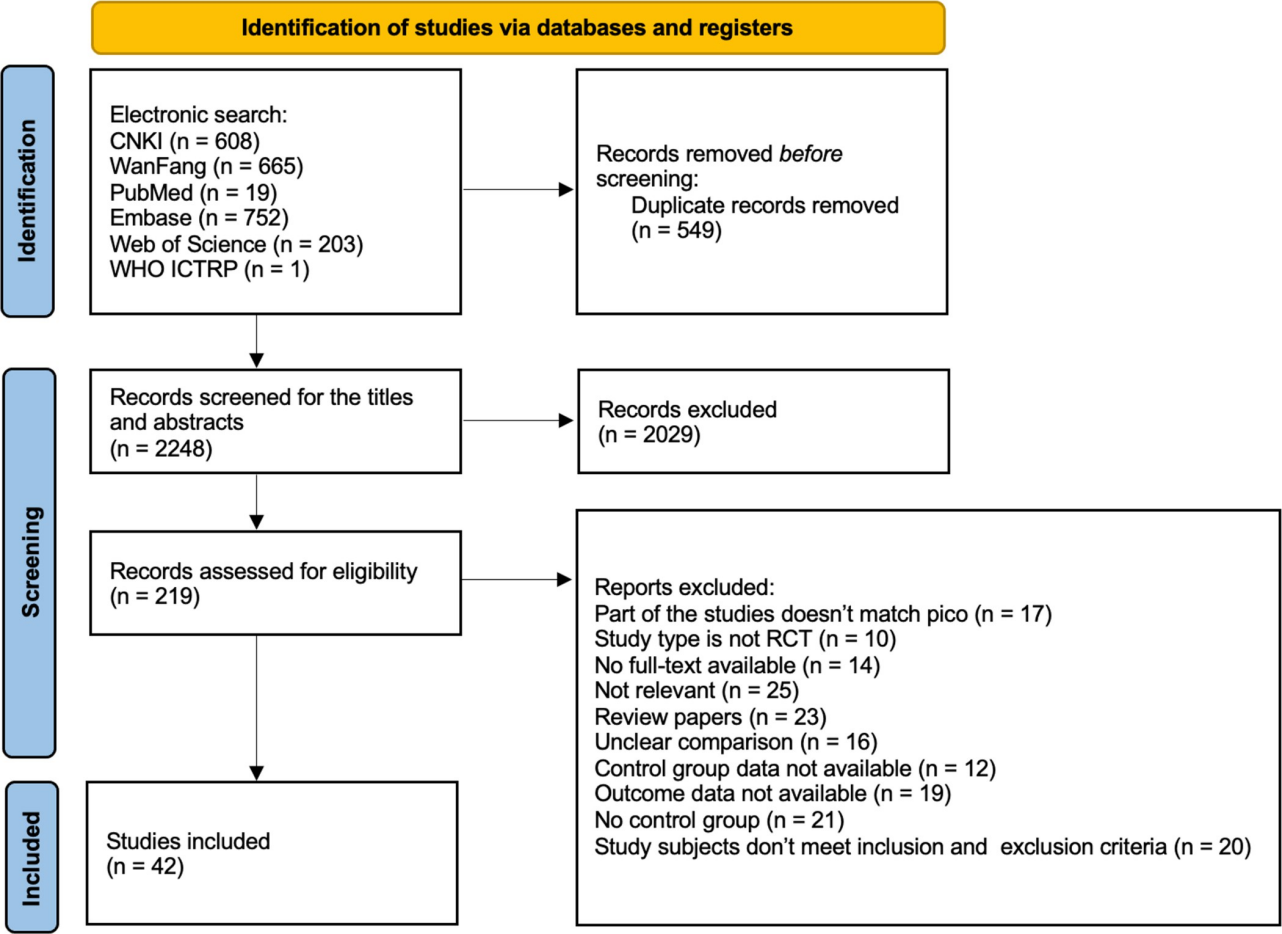
Flow diagram for selection of eligible studies included in the meta-analysis.

A total of 2797 records were identified through database searches. There were 549 duplicates removed electronically, leaving 2248 unduplicated records. After discarding 2029 for not meeting the eligibility criteria according to titles and abstracts, we reviewed the full text of 219 articles for eligibility. Then, we excluded 177 and finally included 42 articles. A PRISMA flowchart was reported in Fig 1.

### Characteristics of included studies

The characteristics of the included studies are summarised in Table 1. Among the 42 eligible randomized controlled trials, 32 studies delivered NPS including 57,325 participants. The remaining 10 delivered IPS including 12,801 participants. All the studies involved were published between 2014 and 2023. Among the NPS studies where participants’ age data was available, the average age was about 26.8. The length of follow-up lasted from 6 to 12 months at most. 23 trials noted specific follow-up methods via telephone calls, texts, WeChat and outpatient service. All controls were selected for routine care. 24 studies calculated the rate of effective contraceptive use, 27 for repeat abortion rate, 13 for follow-up rate and five for patient satisfaction rate.

**Table 1:** Characteristics of all included studies.

Study	Intervention	Study design	Sample size		Mean Age	Length of follow-up	Follow-up methods*	Loss to follow-up*		Outcomes*
			T	C				T	C	
**LiuY 2017**	NPS	RCT	519	527	/	1、3、6、12 m	A	81	261	a, b, c
**WangX 2020**	NPS	RCT	5984	5896	33.1	12 m	A, C	/	/	a
**LiuJ 2015**	NPS	RCT	300	300	20.5	1、3、6、9、12 m	A, B	/	/	b
**JiW 2019**	NPS	RCT	166	170	24	1、6、12 m	A, B	/	/	a, b
**HouC 2016**	NPS	RCT	426	385	30.3	1、3、6、12 m	A	/	/	a, b
**ChenJ 2015**	NPS	RCT	307	304	28.3	6 m	A	134	123	b, c
**MengF 2023**	NPS	RCT	200	200	25.4	1、3、6 m	/	/	/	a, b, d
**JiangL 2020**	NPS	RCT	150	150	/	1、3、6、12 m	A	13	59	b, c
**ZhouY 2015**	NPS	RCT	350	350	25.9	1、3、6、12 m	/	23	35	a, b, c
**ZhouC 2020**	NPS	RCT	320	320	/	14d、1、3、6、12 m	/	146	213	a, b, c
**LiangH 2021**	NPS	RCT	1250	1250	30.6	1、3、6 m	A, C, D	/	/	a
**FengW 2016**	NPS	RCT	375	388	24.4	1、3、6、12 m	A	/	/	a, b
**ChenX 2015**	NPS	RCT	416	416	27.8	3、6、12 m	A	/	/	a, b
**ChenQ 2016**	NPS	RCT	300	300	23.5	1、3、6、12 m	A, C	55	88	a, b, c
**Liu Y 2016**	NPS	RCT	600	600	26.2	12、24 m	A	/	/	d
**Liu X 2020**	NPS	RCT	9048	9025	26.7	1、3、6 m	A, B	/	/	a, b
**Jin M 2016**	NPS	RCT	476	492	27.1	1、3、6、12 m	A, B	/	/	a, b
**Zhang H 2018**	NPS	RCT	712	336	28.2	1、3、6 m	A, B	/	/	a, b
**Wang X 2019**	NPS	RCT	1032	1032	29.4	1、6 m	/	14	18	a, b, c
**Zhang Y 2019**	NPS	RCT	999	999	27.5	1、3、6 m	/	/	/	a, b
**Wang Y 2020**	NPS	RCT	1000	1000	/	14 d、1、3、6、12 m	/	322	796	a, b, c
**Jin X 2015**	NPS	RCT	266	252	23.2	1、3、6 m	A	60	55	b, c
**Chen Z 2014**	NPS	RCT	718	408	30	1、3、6 m	A	/	/	b
**Tang K 2015**	NPS	RCT	500	850	29.5	1、3、6、12 m	A	183	497	a, b, c, d
**Tan L 2020**	NPS	RCT	300	300	25.9	1、6、12 m	A	/	/	a, b, d
**Zhang Y 2014**	NPS	RCT	508	483	29.2	6 m	/	/	/	a
**Wei K 2015**	NPS	RCT	300	300	20.8	10 d、1、3、6、12 m	/	249	269	a, b, c
**Gong X 2020**	NPS	RCT	346	222	26.1	12 m	A, B, C	/	/	a
**Zhang J 2022**	NPS	RCT	200	200	29.4	1、3、6 m	/	/	/	a, b
**Cui C 2015**	NPS	RCT	491	491	25	1、3、6、12 m	A	57	83	b, c
**Wang H 2012**	NPS	RCT	300	300	/	12 m	A	/	/	b, d
**Guo L 2018**	NPS	RCT	110	110	24.7	1、3、6、12 m	A, C, D	22	27	a, b, c
**Xie J 2020**	IPS	RCT	228	228	/	6 m	C, E	6	18	a, c
**Wang J 2017**	IPS	RCT	299	318	18	1、3、6、12 m	A, B, C, G	69	99	a, c
**Cheng X 2022**	IPS	RCT	132	132	20.3	6 m	A, B, C	/	/	a, b
**Sun T 2020**	IPS	RCT	150	150	31.3	1、3、6、12 m	A, C	15	39	a, c
**Li W 2021**	IPS	RCT	300	300	28.2	/	/	/	/	a, d
**Qian S 2019**	IPS	RCT	1752	1752	/	1、3、6 m	A, C	378	709	a, c
**Wu S 2018**	IPS	RCT	1694	1521	26.8	1、3、6、12 m	A, B, C	400	430	a, b, c
**Li H 2020**	IPS	RCT	120	120	24.8	/	A	/	/	a
**Wang Q 2020**	IPS	RCT	980	1008	22	1、3、6 m	A, G	106	189	a, b, c
**Qin X 2017**	IPS	RCT	777	840	25	1、3、6、12 m	A, C, E, F	420	534	a, b, c

NOTE: NPS, normal post-abortion care services; IPS, improved post-abortion care services; RCT, randomized controlled trial; m, month; d, day.

*Follow-up methods: A, telephone call; B, outpatient service; C, WeChat; D, text; E, QQ; F, E-mail; G, other Internet applications.

*Loss to follow-up: number of participants lost to follow-up at the end of the study (6 months and 12 months)

*Outcomes: a, efficient contraceptive use during 1 year (including postoperation, 1, 3, 6 and 12 months); b, repeat abortion rate during 1 year (including 1, 3, 6 and 12 months); c, follow-up rate during 1 year (including 1, 3, 6 and 12 months); d, patient satisfaction.

Among another package, IPS studies, the average age was about 24.5 years old according to eight studies. The distribution of follow-up period was from 1 to 12 months. Compared with NPS studies, one of the improved areas is focused on follow-up methods including social applications and other Internet approaches. Another improved area was forms of intervention such as online and interactive education while humanistic concerns were emphasised. There were 10 studies that calculated the rate of effective contraceptive use, four for repeat abortion rate, seven for follow-up rate and one for patient satisfaction rate.

### Risk of bias assessment

Of the 32 NPS studies, 10 studies were considered high risk of bias and others were given some concerns overall. High risk in randomization process counted in seven studies was due to the possibility allocation sequence was unconcealed, influencing baseline balances. Six studies had a high risk of missing outcome data. All studies had some concerns about deviations from intended interventions and had low risk in the measurement of the outcomes and selection of the reported results (S1 Fig).

Of the 10 IPS studies, four studies had a high risk of overall bias and others had some concerns. Two studies had a high risk of missing outcome data. As same as NPS studies, all studies had some concerns about deviations from intended interventions and had low risk in the measurement of the outcomes and selection of the reported results (S2 Fig).

### Outcomes for NPS studies

#### Effective contraceptive use

**Fig 2:**
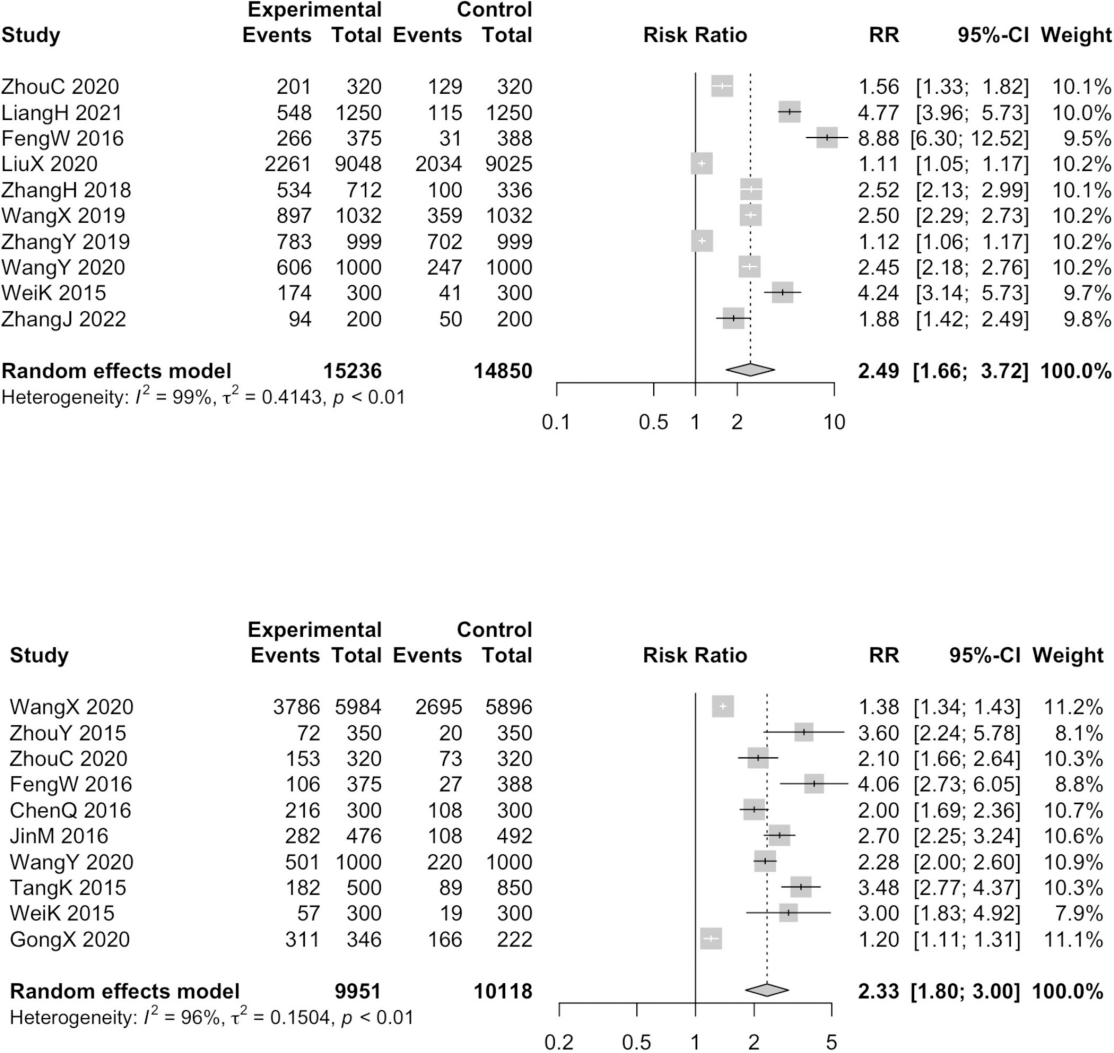
Forest plots for rate of effective contraceptive use in 6 months (A) and 12 months (B) of studies in which normal post-abortion care services were as intervention; (A) top; (B) bottom.

Utilising the random effects model, significant effectiveness was shown in postoperation (RR = 2.86, 95%CI = 1.97–4.16, I^2^ = 98%, 11 studies, 10295 participants) (S3 Fig), 1 month (RR = 2.73, 95%CI = 1.75–4.27, I^2^ = 99%, 5 studies, 5960 participants) (S4 Fig), 3 months (RR = 3.75, 95%CI = 2.05–6.85, I^2^ = 98%, 5 studies, 6503 participants) (S5 Fig), 6 months (RR = 2.49, 95%CI = 1.66–3.72, I^2^ = 99%, 10 studies, 30086 participants) (Fig 2A), 12 months (RR = 2.33, 95%CI = 1.80–3.00, I^2^ = 96%, 10 studies, 20069 participants) (Fig 2B). Overall, the intervention could apparently improve women’s effective contraceptive use at all stages of follow-up.

#### Repeat abortion

**Fig 3:**
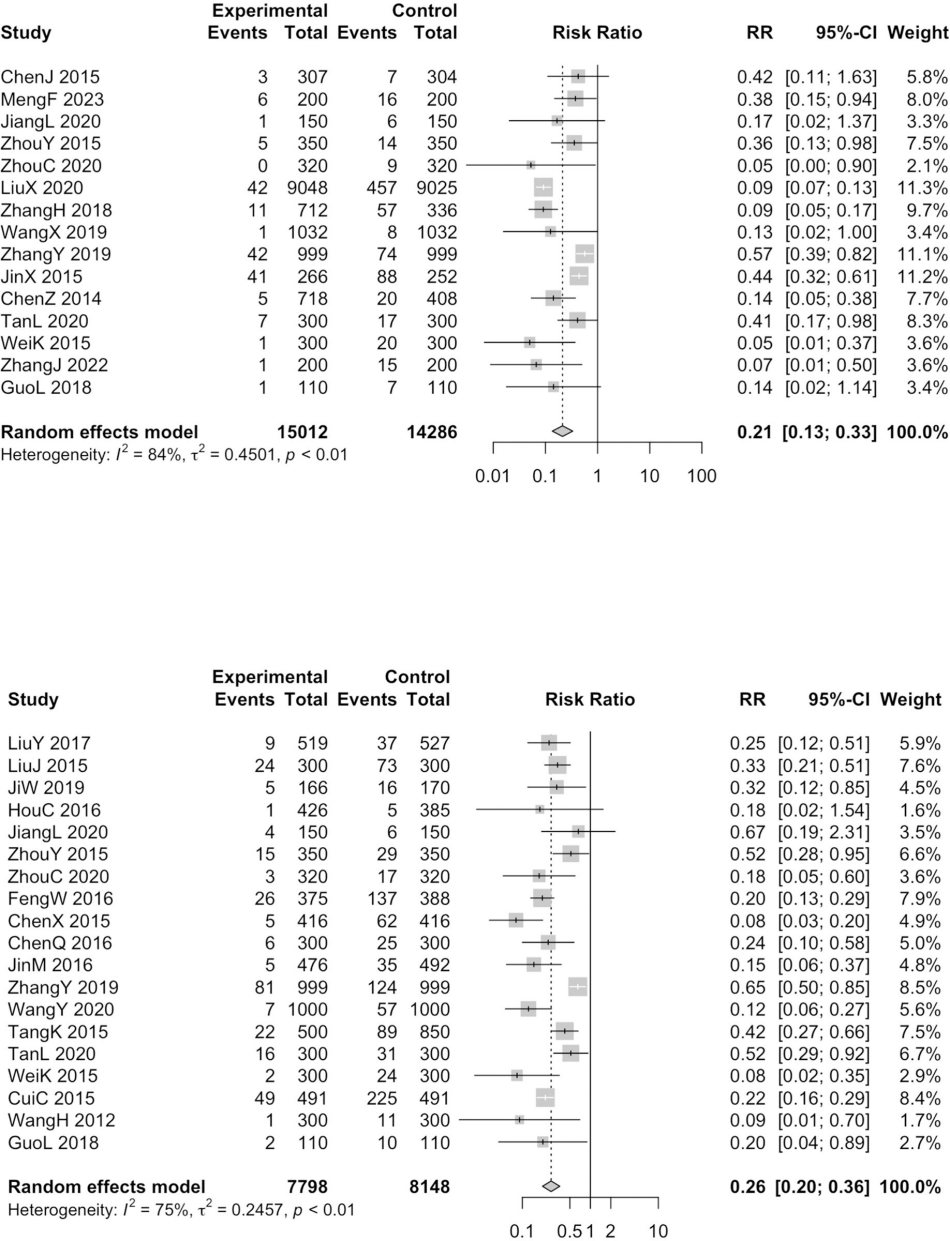
Forest plots for repeat abortion rate in 6 months (A) and 12 months (B) of studies in which normal post-abortion care services were as intervention; (A) top; (B) bottom.

One-and-three-month repeat abortion rate effect estimates were not calculated because of sparse data (zero events for at least one of the groups in the trial). Significant protective effectiveness was revealed in 6 months (RR = 0.21, 95%CI = 0.13–0.33, I^2^ = 84%, 15 studies, 29298 participants) (Fig 3A), 12 months (RR = 0.26, 95%CI = 0.20–0.36, I^2^ = 75%, 19 studies, 15946 participants) (Fig 3B) in the random effects model. It turned out the intervention could effectively reduce women’s repeat abortion rate in the medium and long term.

#### Follow-up

It showed no significance in one-month follow-up rate (RR = 1.19, 95%CI = 1.00–1.42, I^2^ = 98%, 5 studies, 3760 participants) (S6 Fig) which is possibly attributed to the short term. In contrast, it showed modest significance in 3-month period (RR = 1.37, 95%CI = 1.06–1.75, I^2^ = 98%, 5 studies, 4058 studies) (S7 Fig), 6-months (RR = 1.37, 95%CI = 1.03–1.83, I^2^ = 99%, 9 studies, 8303 participants) (S8 Fig), 12 months (RR = 1.46, 95%CI = 1.17–1.82, I^2^ = 98%, 10 studies, 8438 participants) (S9 Fig). These findings underscored the significance of evaluating the medium and long-term effects of the intervention.

#### Patient satisfaction

A minority of hospitals concentrate on this indicator currently. It appeared that PAC services would increase patient satisfaction (RR = 1.15, 95%CI = 1.07–1.24, I^2^ = 93%, 5 studies, 4350 participants) (S10 Fig).

### Outcomes for IPS studies

#### Effective contraceptive use

**Fig 4:**
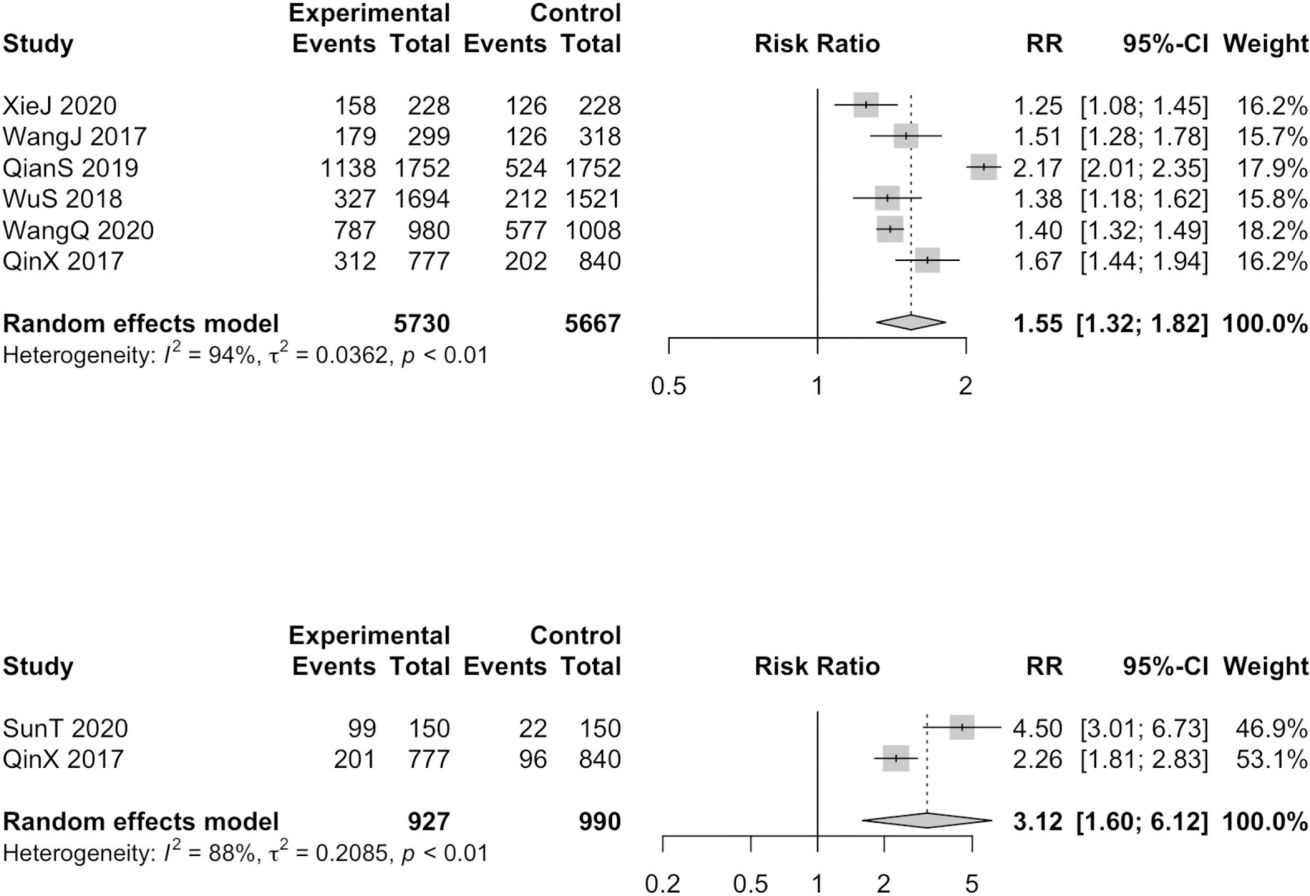
Forest plots for rate of effective contraceptive use in 6 months (A) and 12 months (B) of studies in which improved post-abortion care services were as intervention; (A) top; (B) bottom.

It showed nonsignificant effectiveness in postoperation (RR = 1.43, 95%CI = 0.95–2.15, I^2^ = 94%, 6 studies, 6763 participants) (S11 Fig), 1 month (RR = 1.05, 95%CI = 0.99–1.11, I^2^ = 74%, 3 studies, 5820 participants) (S12 Fig) based on the random effects model. Differently, significant effectiveness was shown in 3 months (RR = 1.37, 95%CI = 1.14–1.64, I^2^ = 98%, 5 studies, 10941 participants) (S13 Fig), 6 months (RR = 1.55, 95%CI = 1.32–1.82, I^2^ = 94%, 6 studies, 11397 participants) (Fig 4A), 12 months (RR = 3.12, 95%CI = 1.60–6.12, I^2^ = 88%, 2 studies, 1917 participants) (Fig 4B). Overall, IPS can be more helpful in the medium and long-term effective contraceptive utilization status.

#### Repeat abortion

It showed intervention could significantly reduce the repeat abortion rate in 6 months (RR = 0.31, 95%CI = 0.19–0.52, I^2^ = 0%, 3 studies, 5467 participants) (S14 Fig), 12 months (RR = 0.25, 95%CI = 0.09–0.75, 1 study, 1617 participants). Results for other periods can’t be synthesised because of sparse data.

#### Follow-up

No significance was found (RR = 1.04, 95%CI = 1.00–1.09, I^2^ = 93%, 5 studies, 7737 participants) (S15 Fig) in one-month period, based on the random effects model. Differently, it suggested significance in 3 months (RR = 1.07, 95%CI = 1.03–1.12, I^2^ = 85%, 6 studies, 11241 participants) (S16 Fig), 6 months (RR = 1.10, 95%CI = 1.03–1.18, I^2^ = 92%, 7 studies, 11697 participants) (S17 Fig), 12 months (RR = 1.25, 95%CI = 1.14–1.37, I^2^ = 0%, 2 studies, 1917 participants) (S18 Fig). Due to the changing follow-up methods such as social applications, the efficiency of follow-up visits has been strengthened.

#### Patient satisfaction

The pooled results showed significance (RR = 1.13, 95%CI = 1.07–1.19, 1 study, 600 participants).

### Sensitivity analysis

Leave-one-out influence analyses revealed that the findings of the meta-analysis did not rely on a particular study. Then the high-risk-bias studies were excluded to examine the robustness of pooled results. It was found that follow-up rates in NPS studies turned not significant in 3 months (RR = 1.01, 95%CI = 0.93–1.08), 6 months (RR = 1.00, 95%CI = 0.99–1.01), and 12 months (RR = 1.18, 95%CI = 0.99–1.40). Other pooled results showed robustness. The results were summerized in S1 Table.

### Publication bias

Results of Peter’s test indicated that publication bias was not significant (t = 1.76, P_Peter’s_ = 0.1023).

### Certainty of evidence

Evidence of different outcomes at different times was qualified using GRADE in S2 Table. Overall, the rate of effective contraceptive use within operation, repeat abortion rate in 6 months, and patient satisfaction in NPS studies were assessed as high certainty. Other body of evidence grades ranged from very low to moderate.

## Discussion

This systematic review summarised the effectiveness carried out by NPS and IPS, which can increase women’s effective contraceptive use, prolong follow-up periods, reduce repeat abortion and improve patient satisfaction. This is also consistent with similar findings from other countries [10, 26–30]. Successive domestic guidelines were generally similar in service process and content therefore the studies weren’t classified based on it [8, 15].

Given the discontinuation of contraceptive use among participants in the studies, in addition to people’s lack of awareness of contraception, the side effects, reliability and effects on future fertility of some modern contraceptives are probably misunderstood which should be emphasized in health education procedures [7, 31]. Many adolescents and unmarried women would not be willing to use long-acting reversible contraception because of conventional views [13]. Social networks and norms play a key role in shaping attitudes and behaviours towards abortion and contraception [12]. Service providers should help to alleviate women’s worries and concerns. Women’s male partners also have the responsibility to take part in PAC services to protect their reproductive health. Some hospitals have invited women’s partners to take part in education and counselling together [32] and it turned out useful. In the IPS studies, we confirm that improving places including follow-up and counselling methods are useful and these might be prioritized. The Internet permeates people’s daily lives, telephone calls and on-the-spot could be replaced by social applications [33, 34]. Most of those who were seeking PAC services are young women, with a high degree of acceptance of WeChat. Maintaining privacy and interactivity of the consultation is crucial in avoiding the embarrassment that comes with face-to-face counselling. This approach encourages patients to communicate more freely, leading to increased trust in the counsellors and a willingness to alleviate their concerns. Many factors influence patient satisfaction, such as the quality of PAC counselling services, the effectiveness of recommended contraceptives and the expertise of personnel. When women seek help with abortion, they should be given customised services besides needy intervention. Service providers not only receive counselling training that strengthens their person-centred care approach but also combines it with the service process and use of counselling skills [35]. Patients will feel their privacy is respected and concerns addressed promptly [36].

In the included studies, the main effective contraceptive measures adopted by the participants included IUD, COC, and sterilization procedures, which were implemented immediately post-abortion. However, some studies have found that compared to delayed insertion, immediate insertion of effective contraceptives such as IUD significantly increased spontaneous expulsion rate while the initiation rate also increased [37]. Another study comparing immediate and delayed insertion of implants has found that immediate insertion resulted in a higher use rate and lower rate of unintended pregnancy, with no significant difference in adverse effects compared to delayed insertion [38]. According to the guidelines [15], it is recommended to immediately insert effective contraceptive measures post-abortion which outweigh any drawbacks.

The included studies evaluated the service effectiveness based on indicators that the researchers themselves were concerned about. Further research is needed to develop a unified quality assessment standard to increase the comparability of PAC services’ effectiveness across different hospitals. In addition, It remains unclear to which extent an increase in the utilization and continuation rates of effective contraceptive measures, as well as a decrease in repeat abortion rate, can be considered indicative of good effectiveness with PAC services. More in-depth research is also needed to explore these aspects.

The limitations of this study were as follows: (1) while it is expected that some respondents may be lost in any study, only a few studies provided specific numbers for these losses. The rate of use of effective contraceptives and repeat abortion cannot be calculated from the number of lost to follow-up participants. (2) we have tried subgroup analysis but given the information available in the included studies, there is no suitable data such as age distribution, marital status, or education level that could be used for subgroup analysis. (3) Some considerable outcomes mentioned in the WHO guidelines such as serious adverse events are lacking in literature screening and data extraction, hence not analyzed. Nevertheless, this systematic review provides a comprehensive synthesis of available evidence on PAC services for Chinese women of childbearing age. Another strength is that evaluations at different phases in a year after abortion procedure allow for immediate, medium and long-term comparison. In addition, this review will have significant implications for designing strategies to improve PAC services to improve contraceptive continuation and maternal reproductive health.

In summary, the present systematic review and meta-analysis consolidates and updates the quantitative effectiveness of over a decade of PAC services implementation in China. PAC services can help increase the rate of effective contraceptive use for women of childbearing age, reduce the incidence of repeat abortion, and at the same time improve follow-up rates and patient satisfaction, thereby protecting women’s fertility. It is also recommended to improve PAC services by service providers. There is a need to explore better strategies to improve PAC services, especially in the aspect of counselling session. Meanwhile, more in-depth research is critical to explore standards for evaluating the quality of PAC services and definition thresholds for effectiveness.

## Supporting information

S1 Checklist PRISMA 2020 checklist. (DOCX)

S1 Appendix Search strategy. (DOCX)

S1 Table Sensitivity analysis results of four outcome indicators after excluding high-risk-bias studies. (DOCX)

S2 Table Grading of recommendations assessment, development and evaluation of evidence for outcomes included in meta-analyses. (DOCX)

S1 Fig Risk of bias for studies in which normal post-abortion care services were as intervention. Green-low risk; yellow-some concerns; red-high risk.
(EPS)

S2 Fig Risk of bias for studies in which improved post-abortion care services were as intervention. Green-low risk; yellow-some concerns; red-high risk.
(EPS)

S3 Fig Forest plot for rate of effective contraceptive use postoperation of studies in which normal post-abortion care services were as intervention. (PNG)

S4 Fig Forest plot for rate of effective contraceptive use in 1 month of studies in which normal post-abortion care services were as intervention. (PNG)

S5 Fig Forest plot for rate of effective contraceptive use in 3 months of studies in which normal post-abortion care services were as intervention. (PNG)

S6 Fig Forest plot for follow-up rate in 1 month of studies in which normal post-abortion care services were as intervention. (PNG)

S7 Fig Forest plot for follow-up rate in 3 months of studies in which normal post-abortion care services were as intervention. (PNG)

S8 Fig Forest plot for follow-up rate in 6 months of studies in which normal post-abortion care services were as intervention. (PNG)

S9 Fig Forest plot for follow-up rate in 12 months of studies in which normal post-abortion care services were as intervention. (PNG)

S10 Fig Forest plot for patient satisfaction rate of studies in which normal post-abortion care services were as intervention. (PNG)

S11 Fig Forest plot of rate of effective contraceptive use postoperation of studies in which improved post-abortion care services were as intervention. (PNG)

S12 Fig Forest plot for rate of effective contraceptive use in 1 month of studies in which improved post-abortion care services were as intervention. (PNG)

S13 Fig Forest plot for rate of effective contraceptive use in 3 month of studies in which improved post-abortion care services were as intervention. (PNG)

S14 Fig Forest plot for repeat abortion rate in 6 month of studies in which improved post-abortion care services were as intervention. (PNG)

S15 Fig Forest plot for follow-up rate in 1 month of studies in which improved post-abortion care services were as intervention. (PNG)

S16 Fig Forest plot for follow-up rate in 3 month of studies in which normal post-abortion care services were as intervention. (PNG)

S17 Fig Forest plot for follow-up rate in 6 month of studies in which normal post-abortion care services were as intervention. (PNG)

S18 Fig Forest plot for follow-up rate in 12 month of studies in which normal post-abortion care services were as intervention. (PNG)
