# Supplementary material for: Effectiveness of post-abortion care services to protect women’s fertility in China: A systematic review with meta-analysis
Source: PLoS One. 2024 Jun 10;19(6):e0304221. doi: 10.1371/journal.pone.0304221 (PMC11164405; doi:10.1371/journal.pone.0304221)
Supplement: S1 Appendix — (DOCX) [file pone.0304221.s002.docx]

**S1 Appendix. Search strategy**

PubMed search strategies:

1 "Aftercare"[MeSH Terms] (227,903)

2 "post-abortion"[Title/Abstract]" (1,003)

3 "services"[Title/Abstract] OR "intervention"[Title/Abstract] OR "education"[Title/Abstract] OR "care"[Title/Abstract] OR "counseling"[Title/Abstract] (3,135,577)

4 "Contraception"[MeSH Terms] OR "Contraception Behavior"[MeSH Terms] OR "Contraceptive Agents"[MeSH Terms] OR "Contraceptive Devices"[MeSH Terms] (89,365)

5 #1 OR #2 (228,843)

6 #3 AND #4 AND #5 (296)

7 limit 6 to (clinical trial) (21)

8 limit 7 to (Randomized Controlled Trial) (21)

9 limit 8 to (from 2010 – 2023) (19)

EMBASE search strategies:

1 'aftercare'/exp OR 'aftercare':ab,ti (2,199,126)

2 'post-abortion':ab,ti OR 'post abortion':ab,ti (1315)

3 'services':ab,ti OR 'intervention':ab,ti OR 'education':ab,ti OR 'care':ab,ti OR 'counseling':ab,ti (4,376,600)

4 'contraception'/exp OR 'contraception behavior'/exp OR 'contraceptive agents'/exp OR 'contraceptive devices'/exp (375,153)

5 #1 OR #2 (2,200,258)

6 #3 AND #4 AND #5 (6544)

7 #6 AND ('clinical trial'/de OR 'randomized controlled trial'/de) AND [2010-2023]/py (752)

Web of Science search strategy:

1 TS=(aftercare) (3207)

2 TS=(post-abortion) OR TS=(post abortion) (2733)

3 #1 OR #2 (5937)

4 ((((TS=(services)) OR TS=(intervention)) OR TS=(education)) OR TS=(care)) OR TS=(counseling) (3516392)

5 (((TS=(contraception)) OR TS=(contraception behavior)) OR TS=(contraceptive agents)) OR TS=(contraceptive devices) (27454)

6 #3 AND #4 AND #5 (274)

7 #3 AND #4 AND #5 AND PY=(2010-2023) (231)

8 #3 AND #4 AND #5 AND PY=(2010-2023) AND Article (Document Types)(203)

CNKI and Wanfang search strategy:

(mesh term:("post-abortion care") or mesh term:("post-abortion contraception") or mesh term:("post-abortion contraceptive services")) and (ab:("counselling") or ab:("education") or ab:("intervention")) and publication date:[2011-01-01 to 2023-06-30]
