# Supplementary material for: Effectiveness of post-abortion care services to protect women’s fertility in China: A systematic review with meta-analysis
Source: PLoS One. 2024 Jun 10;19(6):e0304221. doi: 10.1371/journal.pone.0304221 (PMC11164405; doi:10.1371/journal.pone.0304221)
Supplement: S1 Table — (DOCX) [file pone.0304221.s003.docx]

| Outcomes | Number of studies | RR (95% CI) | Heterogeneity | |
| --- | --- | --- | --- | --- |
|  |  |  | **I^2^ (%)** | **p Value** |
| Effective contraceptive  implementation rates | | | | |
| Postoperation | NPS* (n = 10) | 2.68 (1.82-3.94) | 98 | <0.01 |
|  | IPS** (n = 5) | 1.55 (0.96-2.50) | 95 | <0.01 |
| 1 month | NPS (n = 1) | 2.30 (1.73-3.04) |  |  |
|  | IPS (n = 2) | 1.01 (0.99-1.04) | 0 | 0.50 |
| 3 months | NPS (n = 1) | 9.90 (7.21-13.61) |  |  |
|  | IPS (n = 2) | 1.25 (1.20-1.30) | 0 | 0.78 |
| 6 months | NPS (n = 5) | 2.57 (1.33-4.96) | 99 | <0.01 |
|  | IPS (n = 3) | 1.39 (1.31-1.48) | 33 | 0.22 |
| 12 months | NPS (n = 5) | 2.46 (1.67-3.63) | 96 | <0.01 |
|  | / | / | / | / |
| Repeat abortion rates | | | | |
| 6 months | NPS (n = 9) | 0.28 (0.17-0.47) | 73 | <0.01 |
|  | IPS (n = 2) | 0.24 (0.12-0.47) | 0 | 0.76 |
| 12 months | NPS (n = 16) | 0.30 (0.22-0.40) | 76 | <0.01 |
|  | IPS (n = 2) | 0.23 (0.09-0.61) | 0 | 0.74 |
| Follow up rates | | | | |
| 1 month | NPS (n = 1) | 1.02 (0.95-1.10) |  |  |
|  | IPS (n = 2) | 1.01 (1.00-1.03) | 0 | 0.72 |
| 3 months | NPS (n = 1) | 1.01 (0.93-1.08) |  |  |
|  | IPS (n = 2) | 1.07 (1.01-1.14) | 68 | 0.08 |
| 6 months | NPS (n = 3) | 1.00 (0.99-1.01) | 0 | 0.72 |
|  | IPS (n = 3) | 1.08 (1.05-1.12) | 4 | 0.35 |
| 12 months | NPS (n = 5) | 1.18 (0.99-1.40) | 95 | <0.01 |
|  | IPS (n = 3) | 1.23 (1.14-1.34) | 45 | 0.16 |
| Patient satisfaction | NPS (n = 4) | 1.17 (1.07-1.28) | 95 | <0.01 |
|  | IPS (n = 1) | 1.13 (1.07-1.19) |  |  |

S1 Table Sensitivity analysis results of four outcome indicators after excluding high-risk-bias studies

*NPS: Normal post-abortion care services, the procedures include public education, personalized consultation, guidance on the immediate implementation of effective contraceptive measures after abortion, and follow-up at 1, 3, 6, and 12 months post-abortion by telephone call or subsequent visit.

**IPS: Improved post-abortion care services, which go beyond NPS, including but not limited to improvements in service format, content, and timing.
