# Supplementary material for: Effectiveness of post-abortion care services to protect women’s fertility in China: A systematic review with meta-analysis
Source: PLoS One. 2024 Jun 10;19(6):e0304221. doi: 10.1371/journal.pone.0304221 (PMC11164405; doi:10.1371/journal.pone.0304221)
Supplement: S2 Table — (DOCX) [file pone.0304221.s004.docx]

| **Outcomes** | **No. of studies** | **Risk of bias** | **Inconsistency** | **Indirectness** | **Imprecision** | **Publication Bias** | **Certainty**  **of evidence** |
| --- | --- | --- | --- | --- | --- | --- | --- |
| **rate of effective contraceptive use** | | | | | | | |
| Postoperation | NPS* (n = 11) | No | No | No | No | No | High |
|  | IPS** (n = 6) | No | Very Serious | No | Serious | NA | Very low |
| 1 month | NPS (n = 5) | Serious | No | No | No | NA | Medium |
|  | IPS (n = 3) | Serious | Serious | No | Serious | NA | Very low |
| 3 months | NPS (n = 5) | Serious | No | No | No | NA | Medium |
|  | IPS (n = 5) | Serious | No | No | No | NA | Medium |
| 6 months | NPS (n = 10) | Serious | No | No | No | No | Medium |
|  | IPS (n = 6) | Serious | No | No | No | NA | Medium |
| 12 months | NPS (n = 10) | Serious | No | No | No | No | Medium |
|  | IPS (n = 2) | Very serious | No | No | Serious | NA | Very low |
| **Repeat abortion rate** | | | | | | | |
| 6 months | NPS (n = 15) | No | No | No | Serious | No | Medium |
|  | IPS (n = 3) | Serious | Serious | No | Very serious | NA | Very low |
| 12 months | NPS (n = 19) | No | No | No | No | No | High |
|  | IPS (n = 1) | / | / | / | / | / | / |
| **Follow up rate** | | | | | | | |
| 1 month | NPS (n = 5) | Serious | Serious | No | No | NA | Low |
|  | IPS (n = 5) | Serious | Serious | No | Serious | NA | Very low |
| 3 months | NPS (n = 5) | Serious | Serious | No | Serious | NA | Very low |
|  | IPS (n = 5) | Serious | No | No | Serious | NA | Low |
| 6 months | NPS (n = 9) | Serious | Serious | No | No | NA | Low |
|  | IPS (n = 7) | Serious | No | No | No | NA | Medium |
| 12 months | NPS (n = 10) | Serious | Serious | No | Serious | No | Very low |
|  | IPS (n = 2) | Very serious | No | No | No | NA | Low |
| **Patient satisfaction** | | | | | | | |
|  | NPS (n = 5) | No | No | No | No | NA | High |
|  | IPS (n = 1) | / | / | / | / | / | / |

S2 Table Grading of Recommendations Assessment, Development and Evaluation of Evidence for outcomes included in meta-analyses

*NPS: Normal post-abortion care services, the procedures include public education, personalized consultation, guidance on the immediate implementation of effective contraceptive measures after abortion, and follow-up at 1, 3, 6, and 12 months post-abortion by telephone call or subsequent visit.

**IPS: Improved post-abortion care services, which go beyond NPS, including but not limited to improvements in service format, content, and timing.
