# Supplementary figures and images for: Effectiveness of post-abortion care services to protect women’s fertility in China: A systematic review with meta-analysis
Source: PLoS One. 2024 Jun 10;19(6):e0304221. doi: 10.1371/journal.pone.0304221 (PMC11164405; doi:10.1371/journal.pone.0304221)

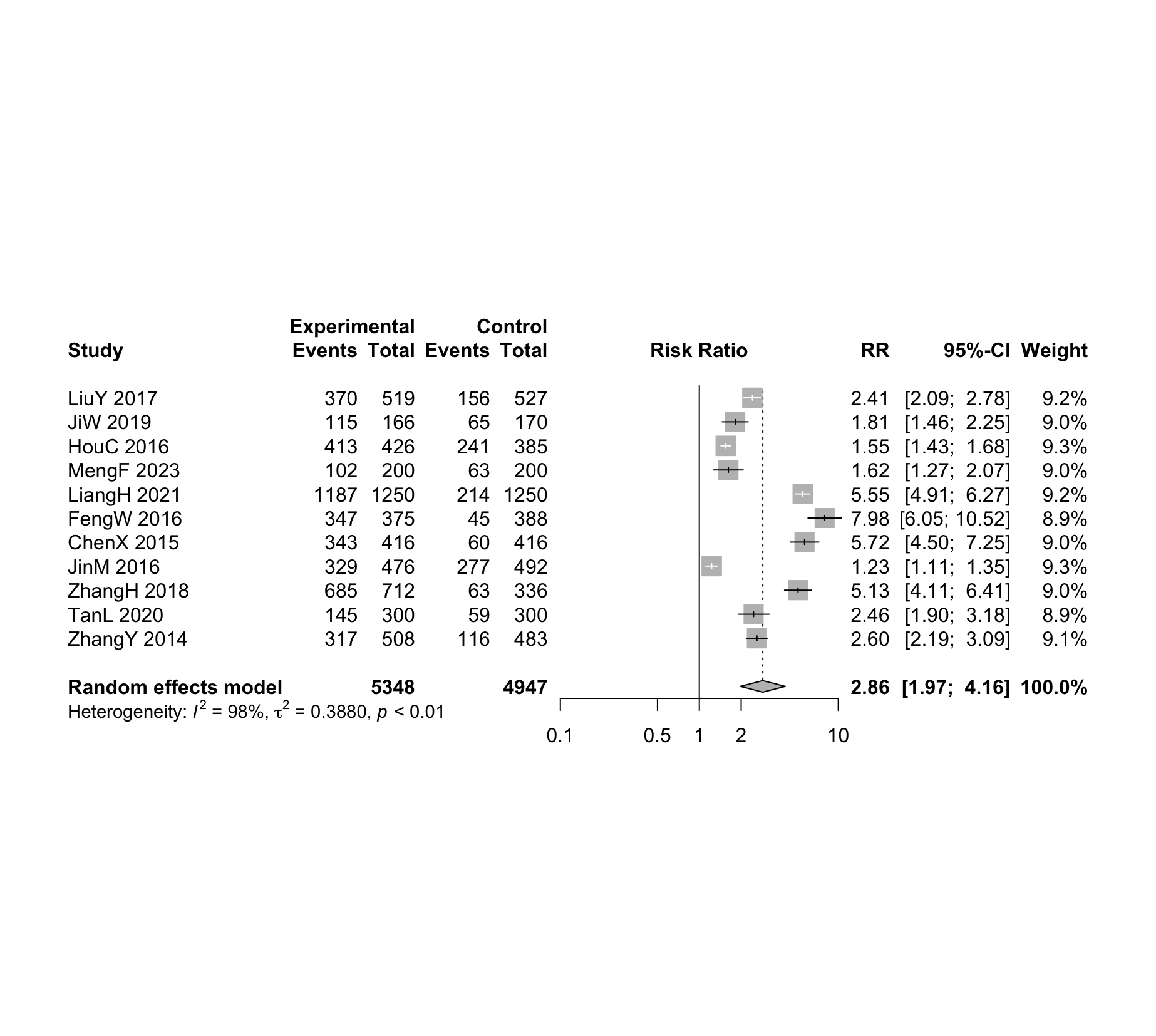

Supplement: S3 Fig — (PNG) [file pone.0304221.s007.PNG]

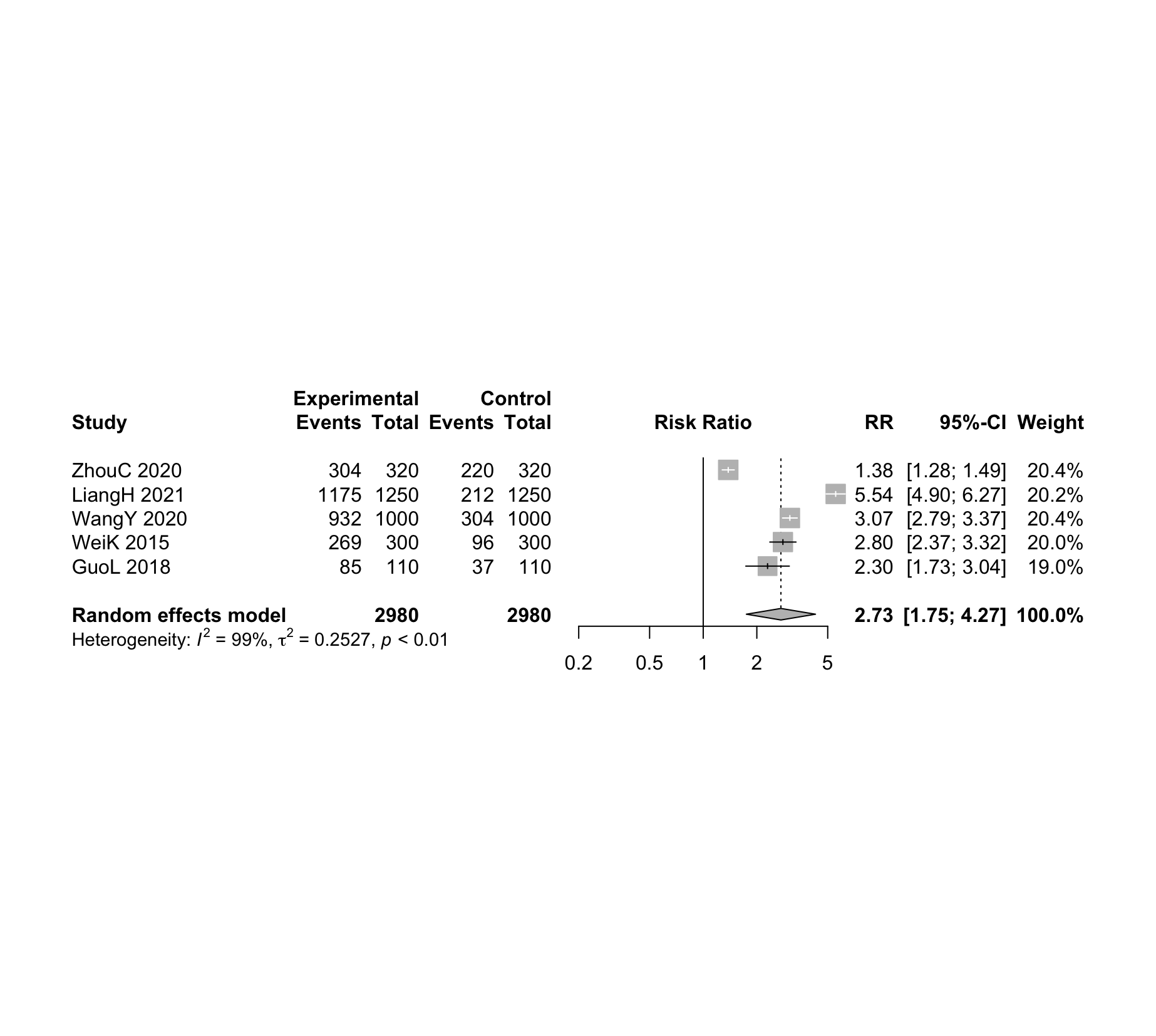

Supplement: S4 Fig — (PNG) [file pone.0304221.s008.PNG]

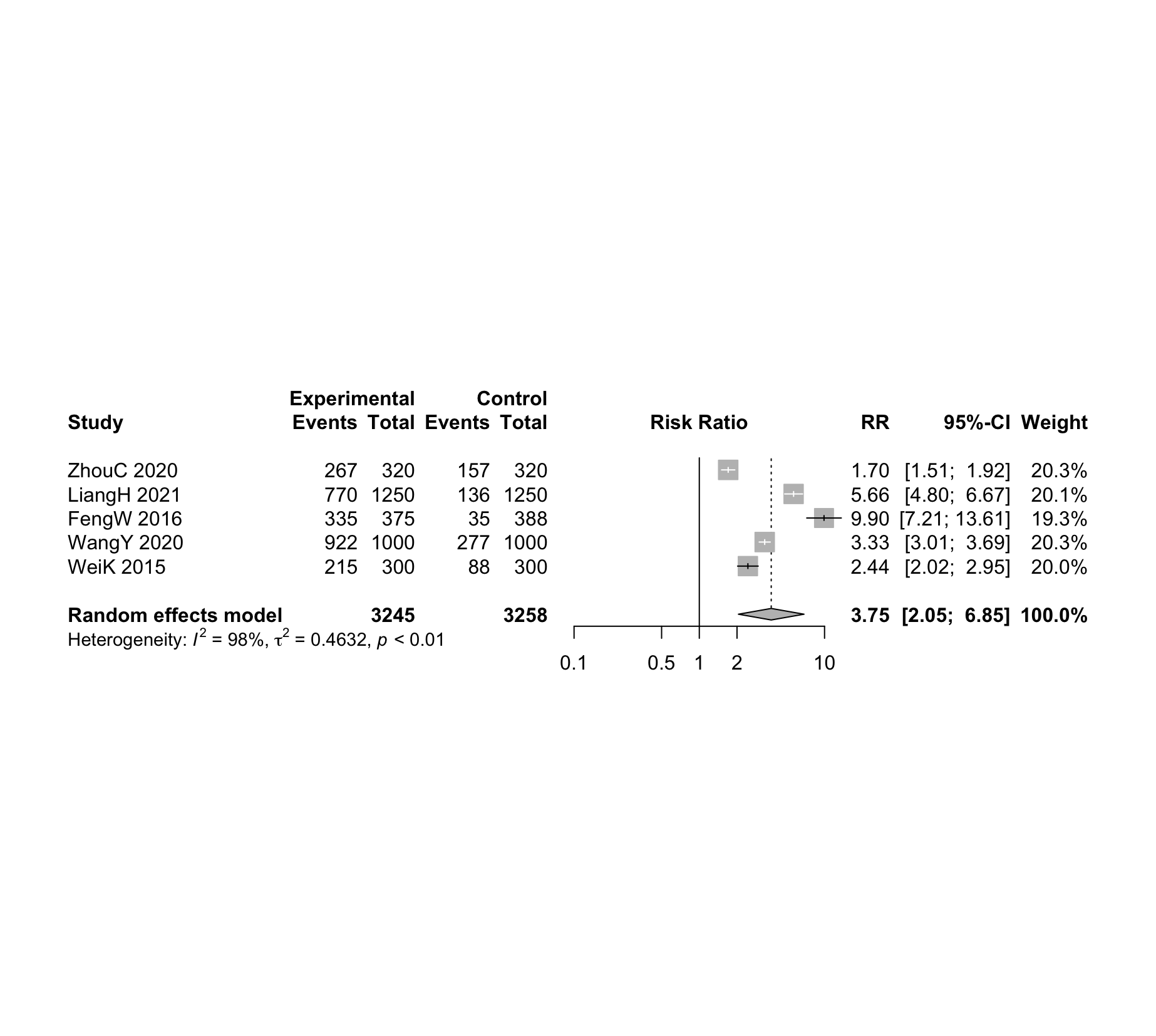

Supplement: S5 Fig — (PNG) [file pone.0304221.s009.PNG]

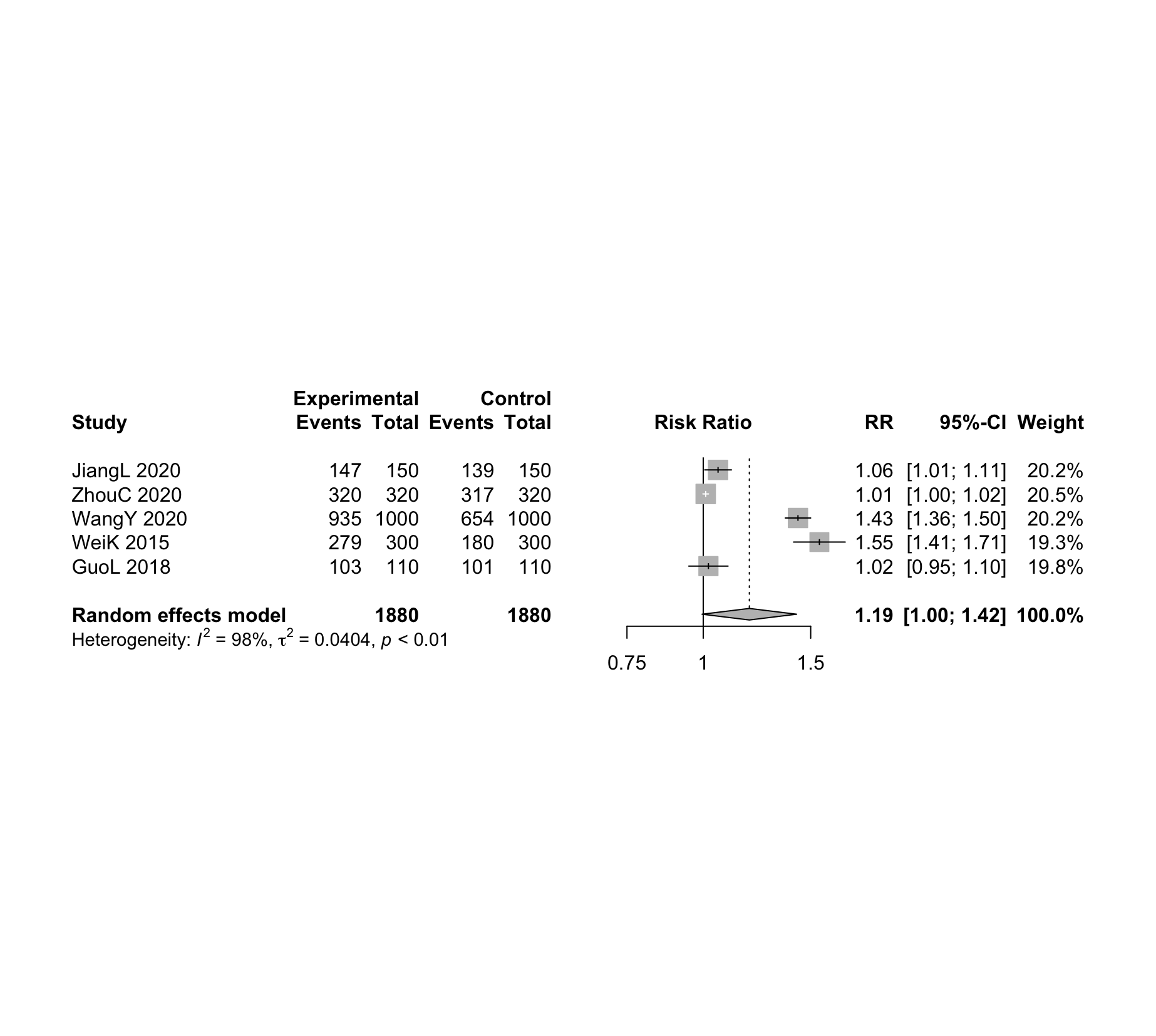

Supplement: S6 Fig — (PNG) [file pone.0304221.s010.PNG]

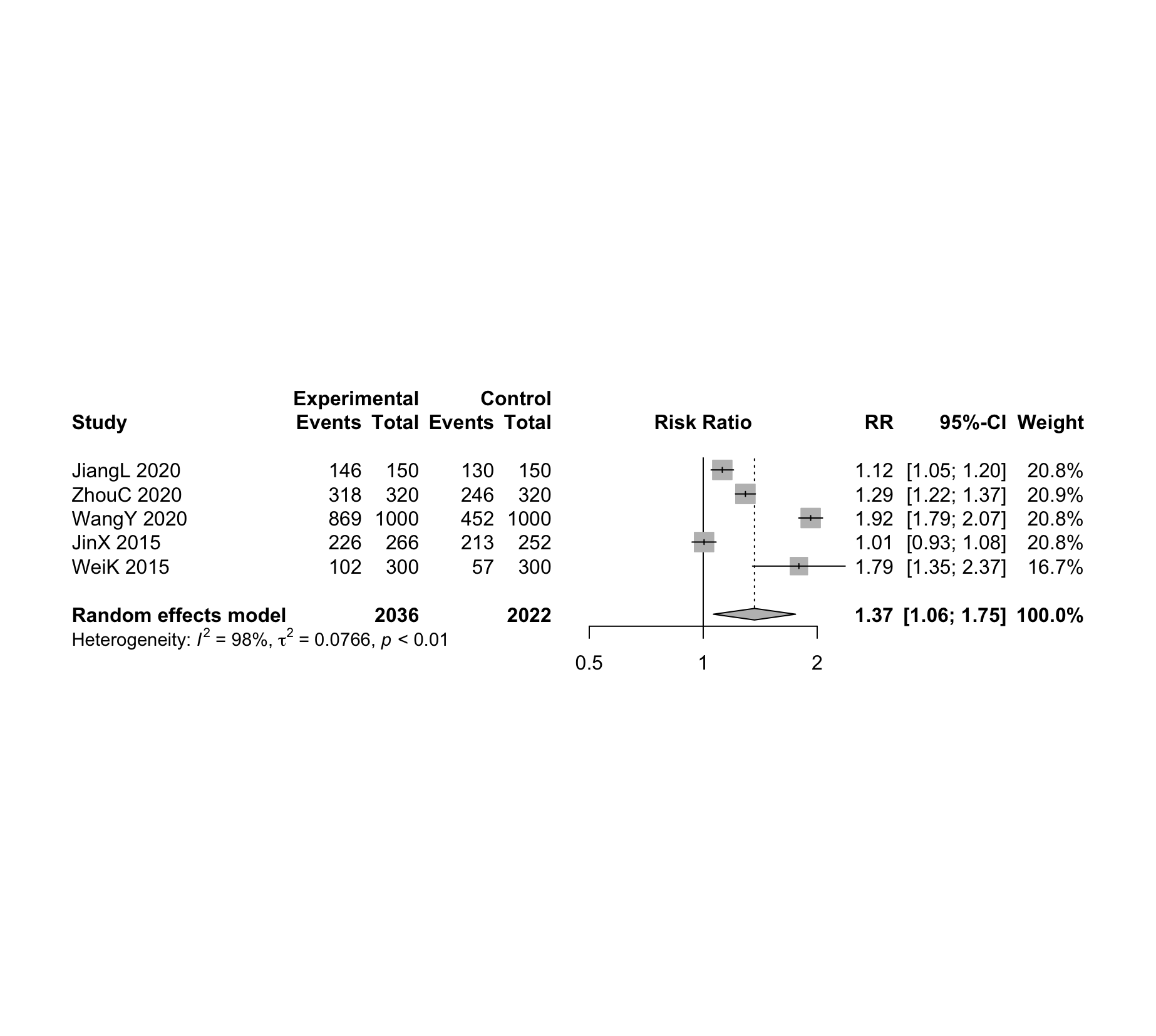

Supplement: S7 Fig — (PNG) [file pone.0304221.s011.PNG]

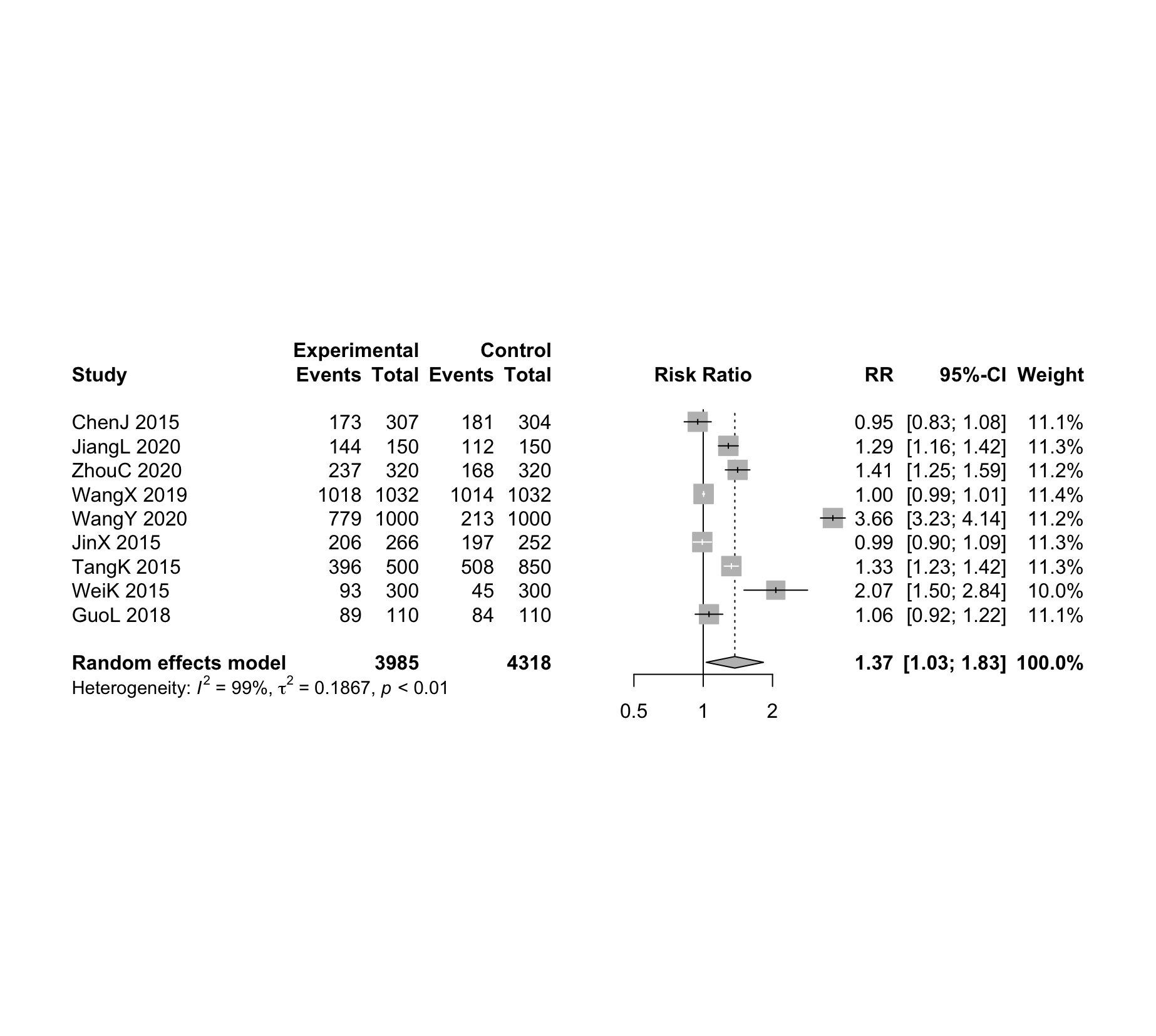

Supplement: S8 Fig — (PNG) [file pone.0304221.s012.PNG]

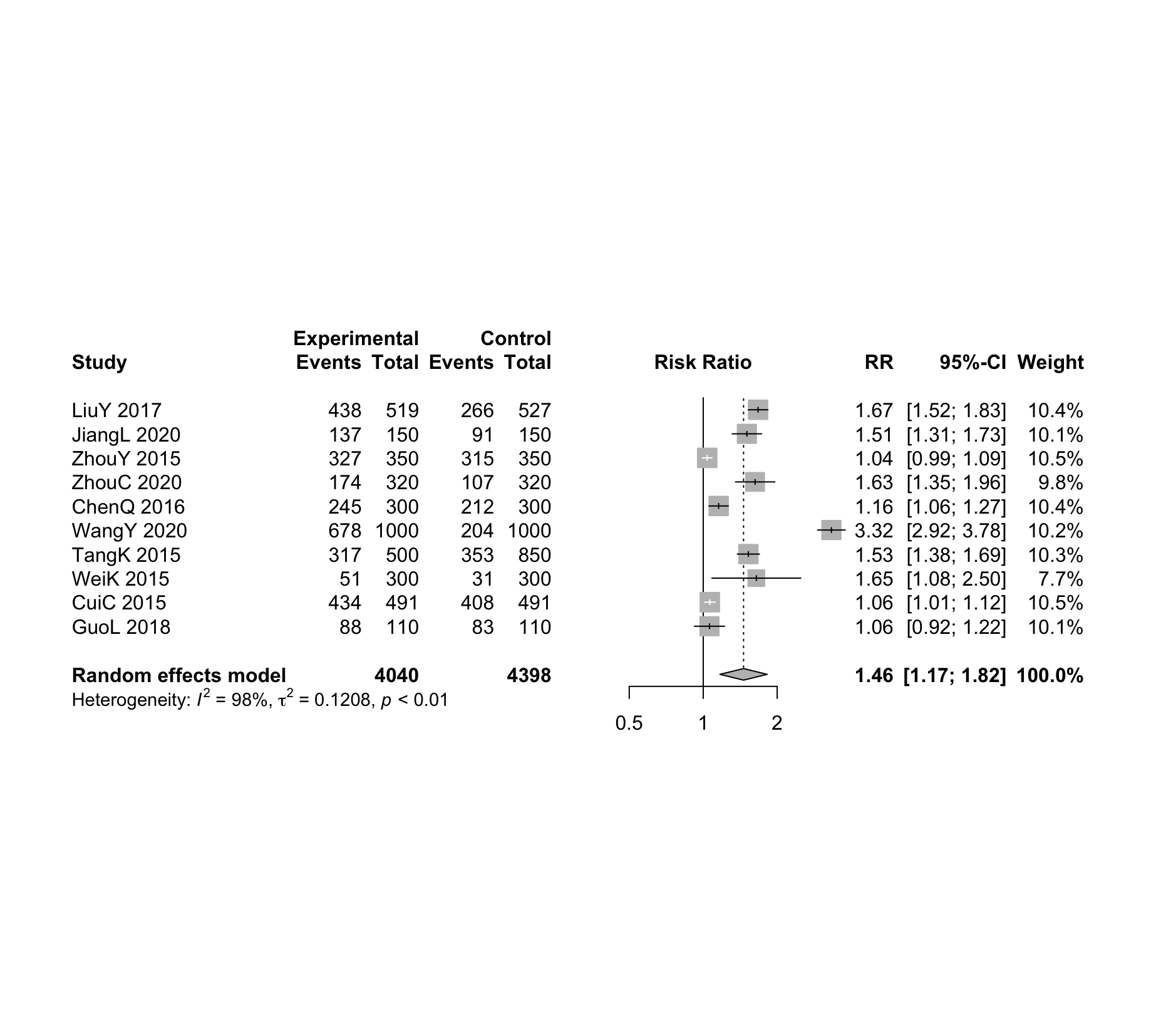

Supplement: S9 Fig — (PNG) [file pone.0304221.s013.PNG]

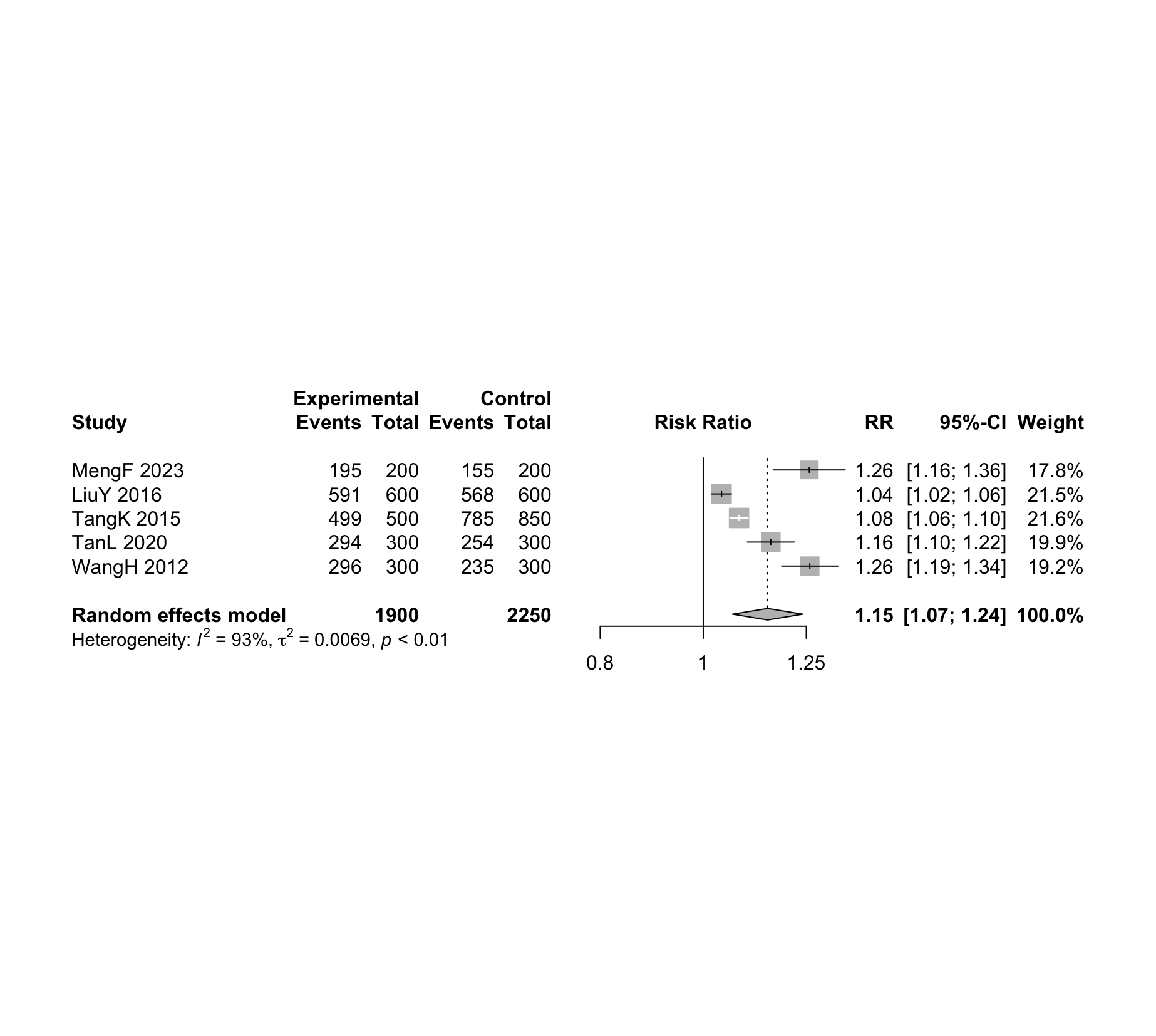

Supplement: S10 Fig — (PNG) [file pone.0304221.s014.PNG]

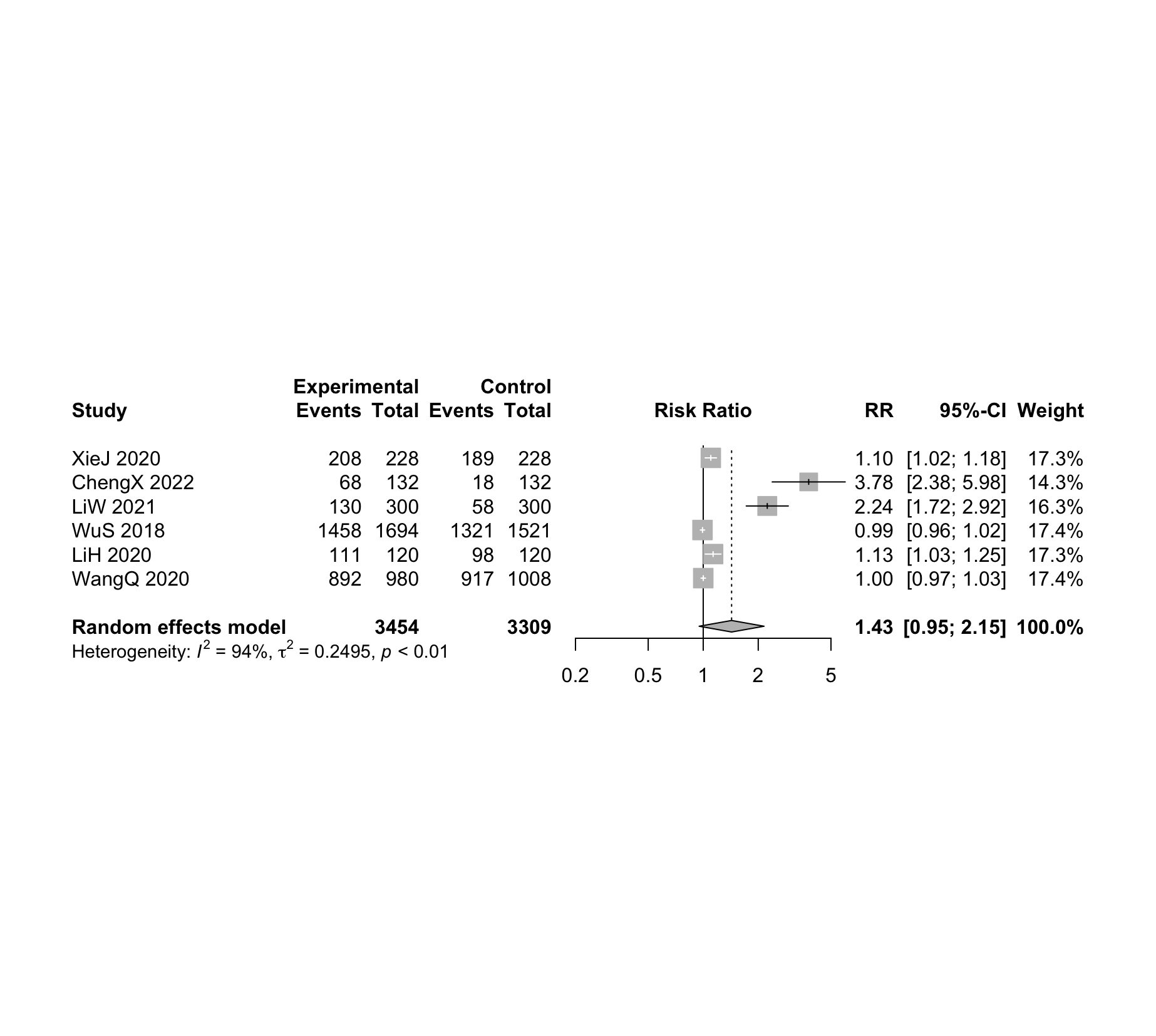

Supplement: S11 Fig — (PNG) [file pone.0304221.s015.PNG]

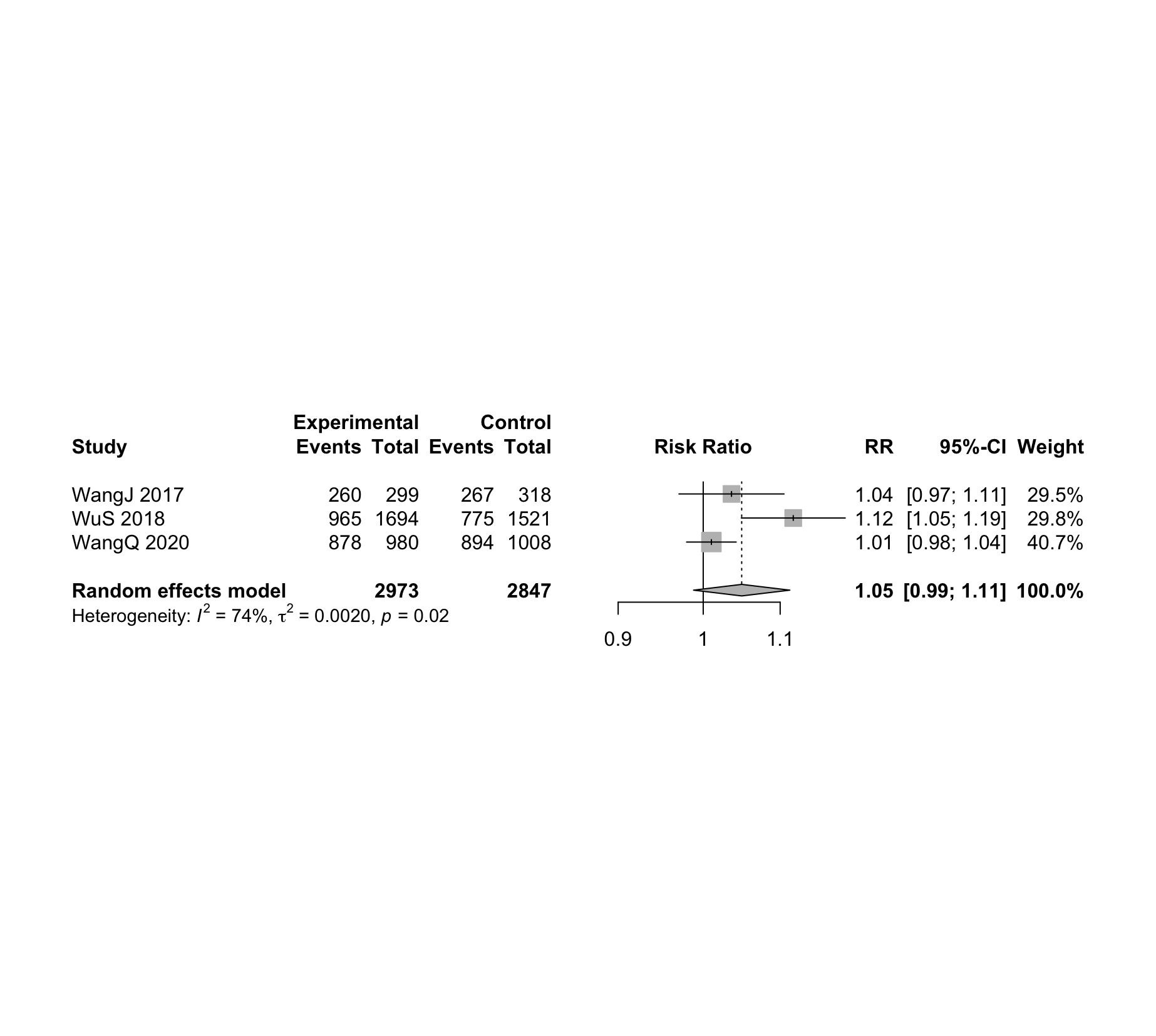

Supplement: S12 Fig — (PNG) [file pone.0304221.s016.PNG]

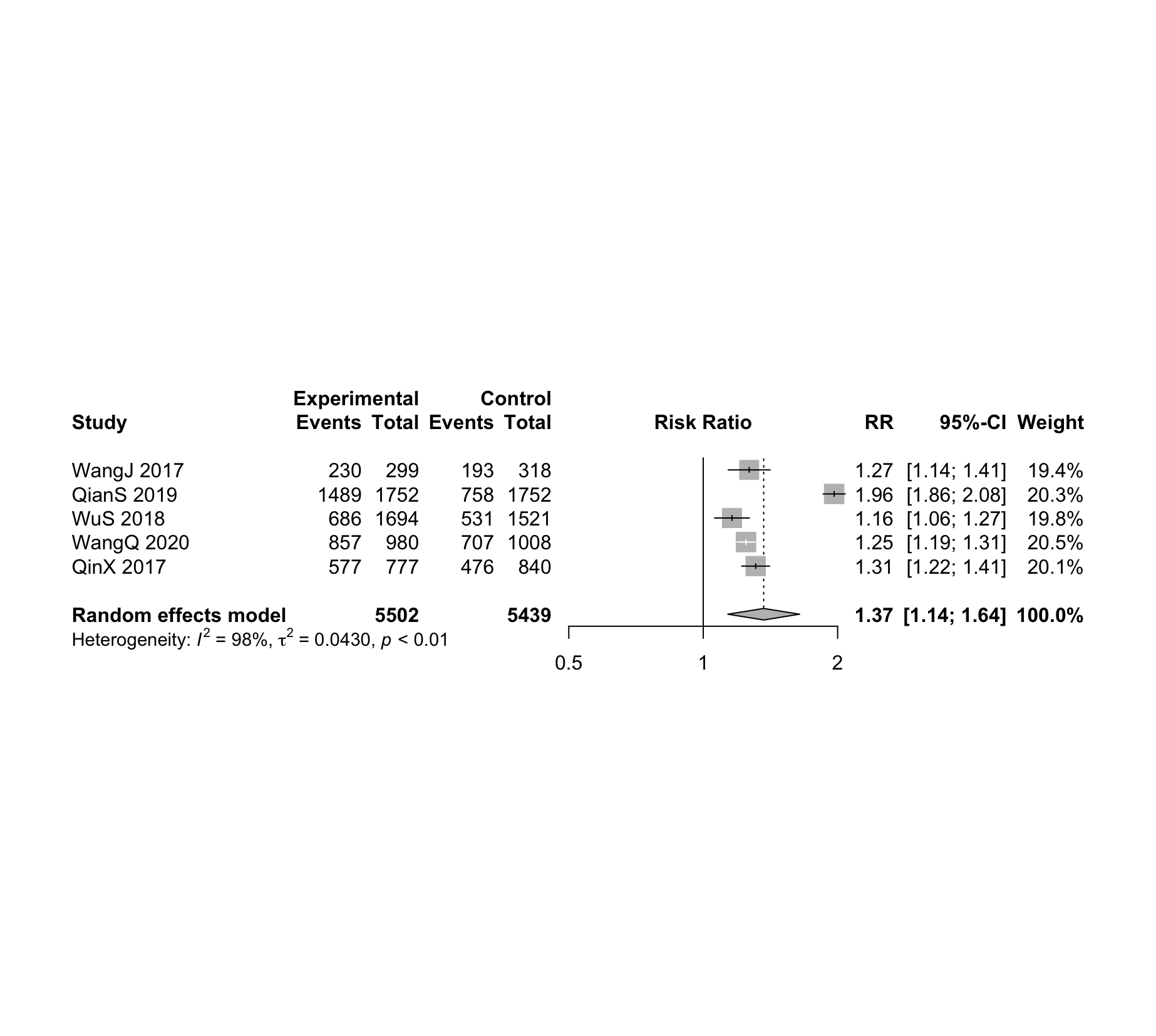

Supplement: S13 Fig — (PNG) [file pone.0304221.s017.PNG]

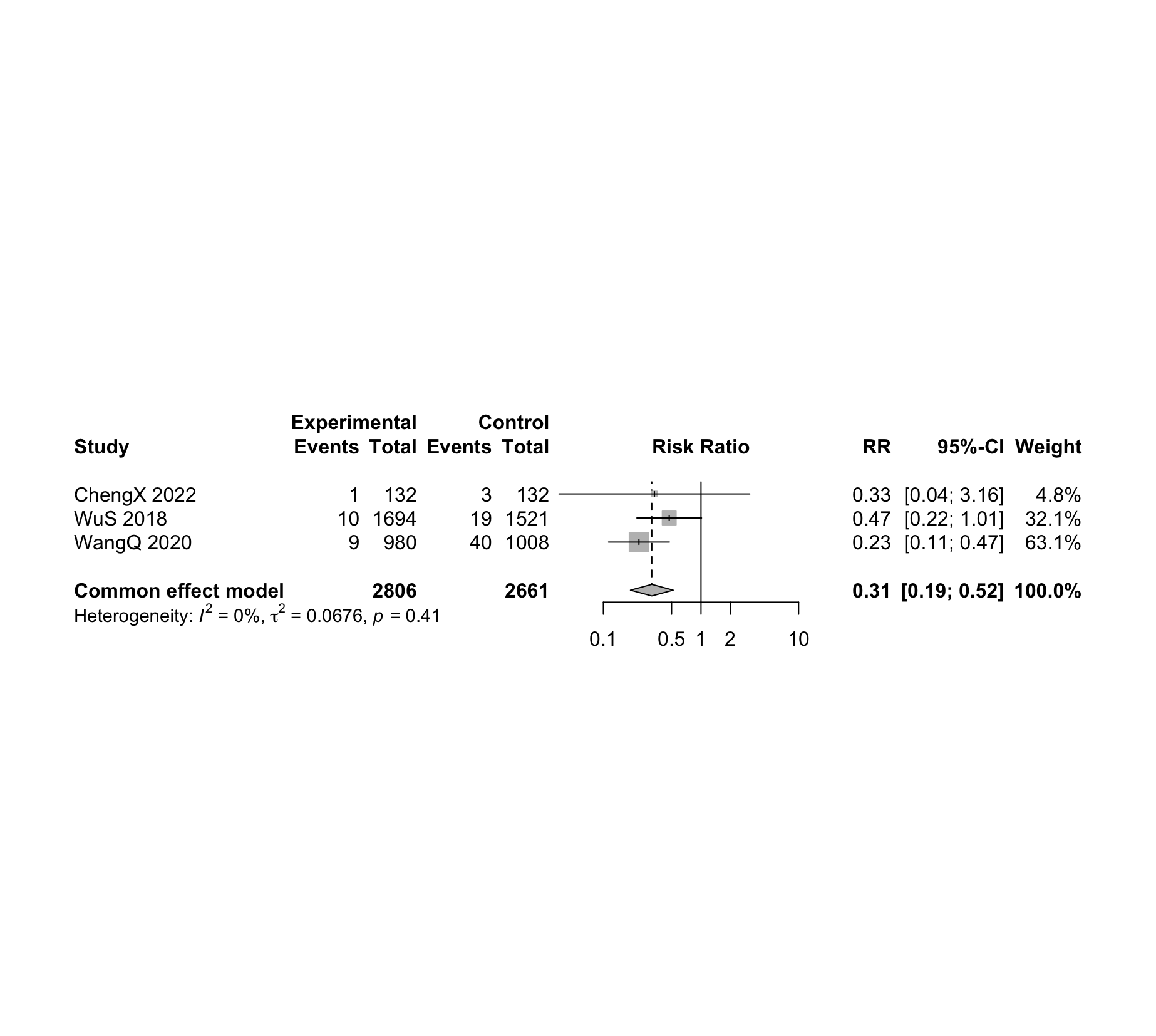

Supplement: S14 Fig — (PNG) [file pone.0304221.s018.PNG]

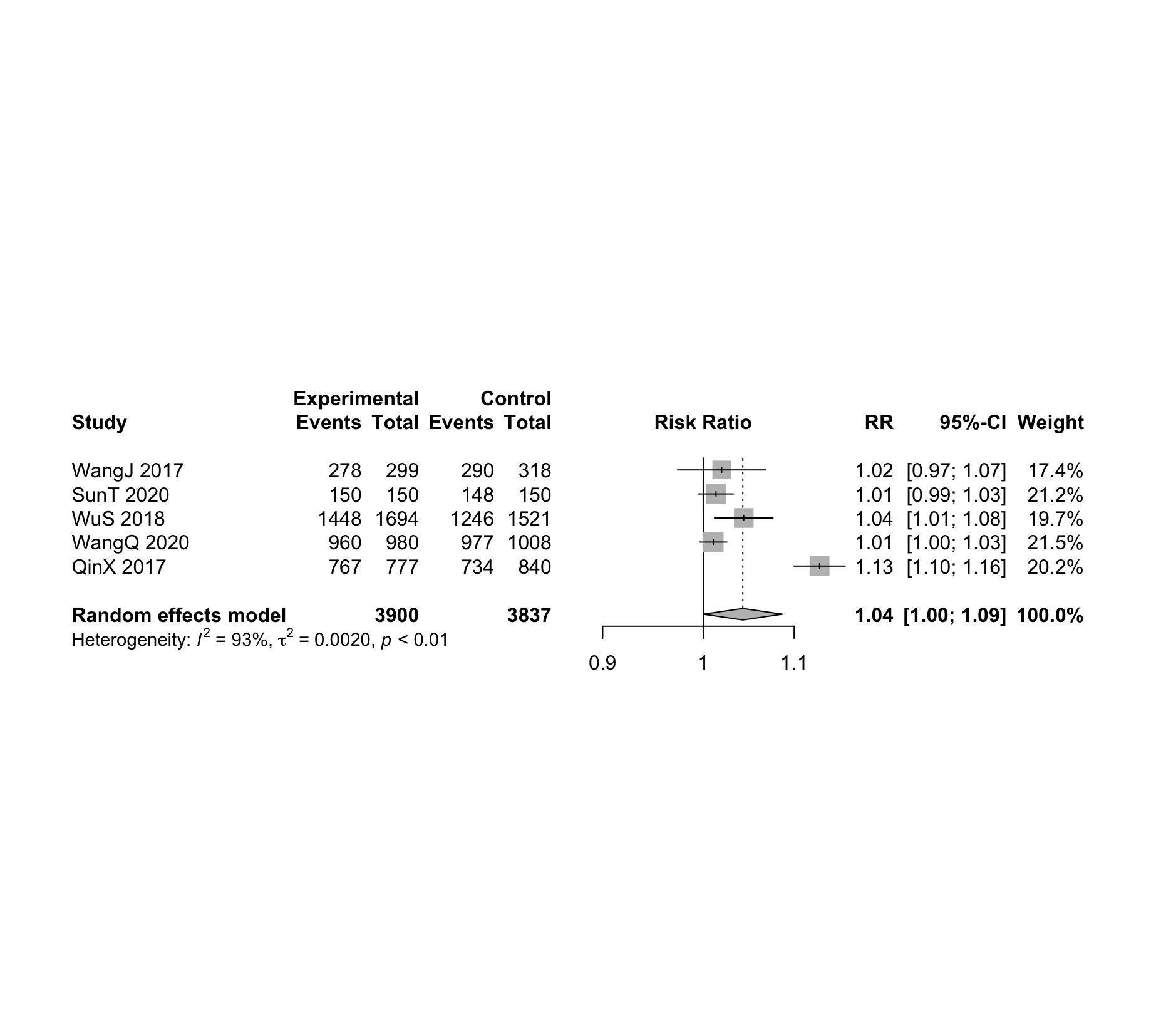

Supplement: S15 Fig — (PNG) [file pone.0304221.s019.PNG]

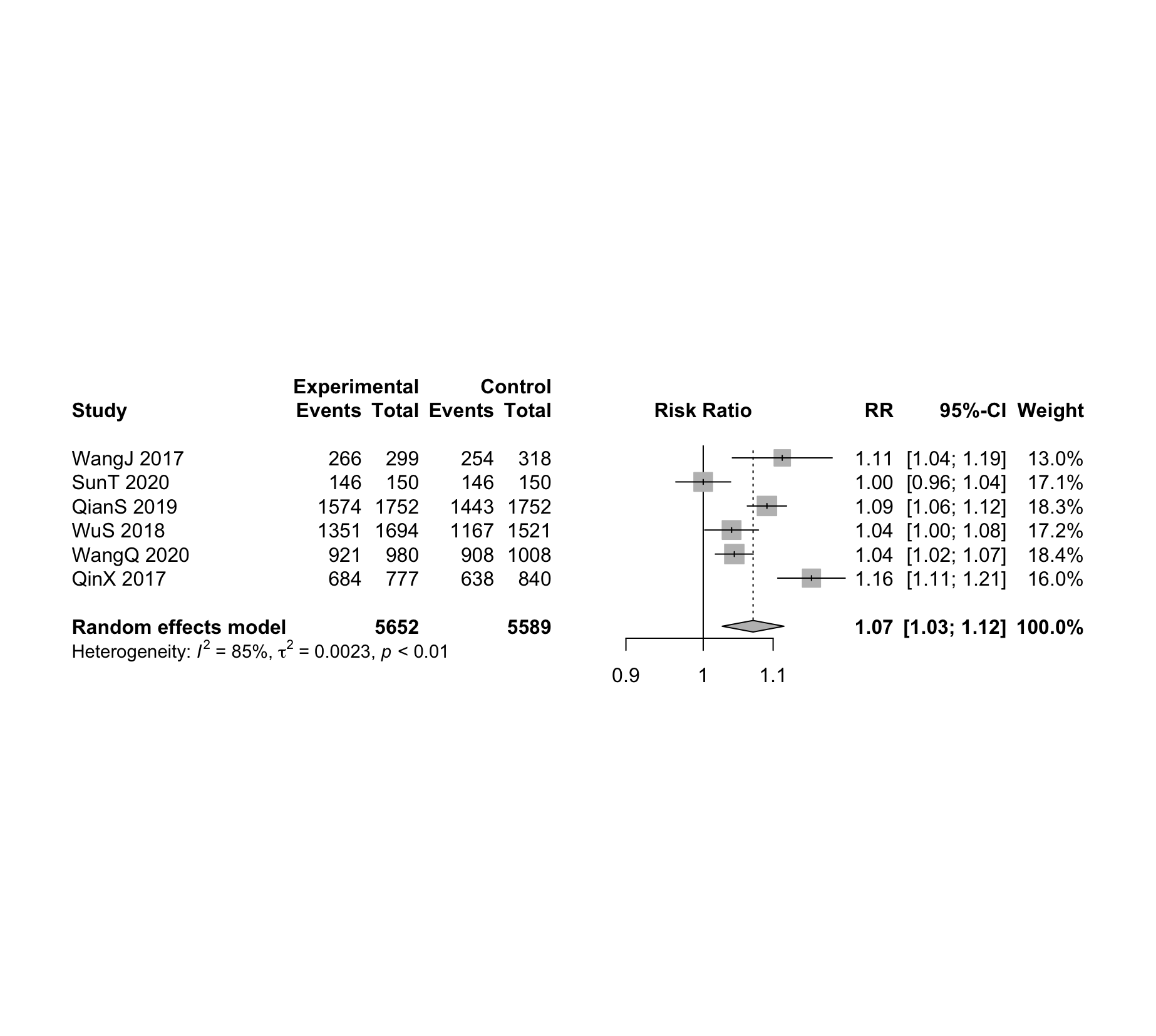

Supplement: S16 Fig — (PNG) [file pone.0304221.s020.PNG]

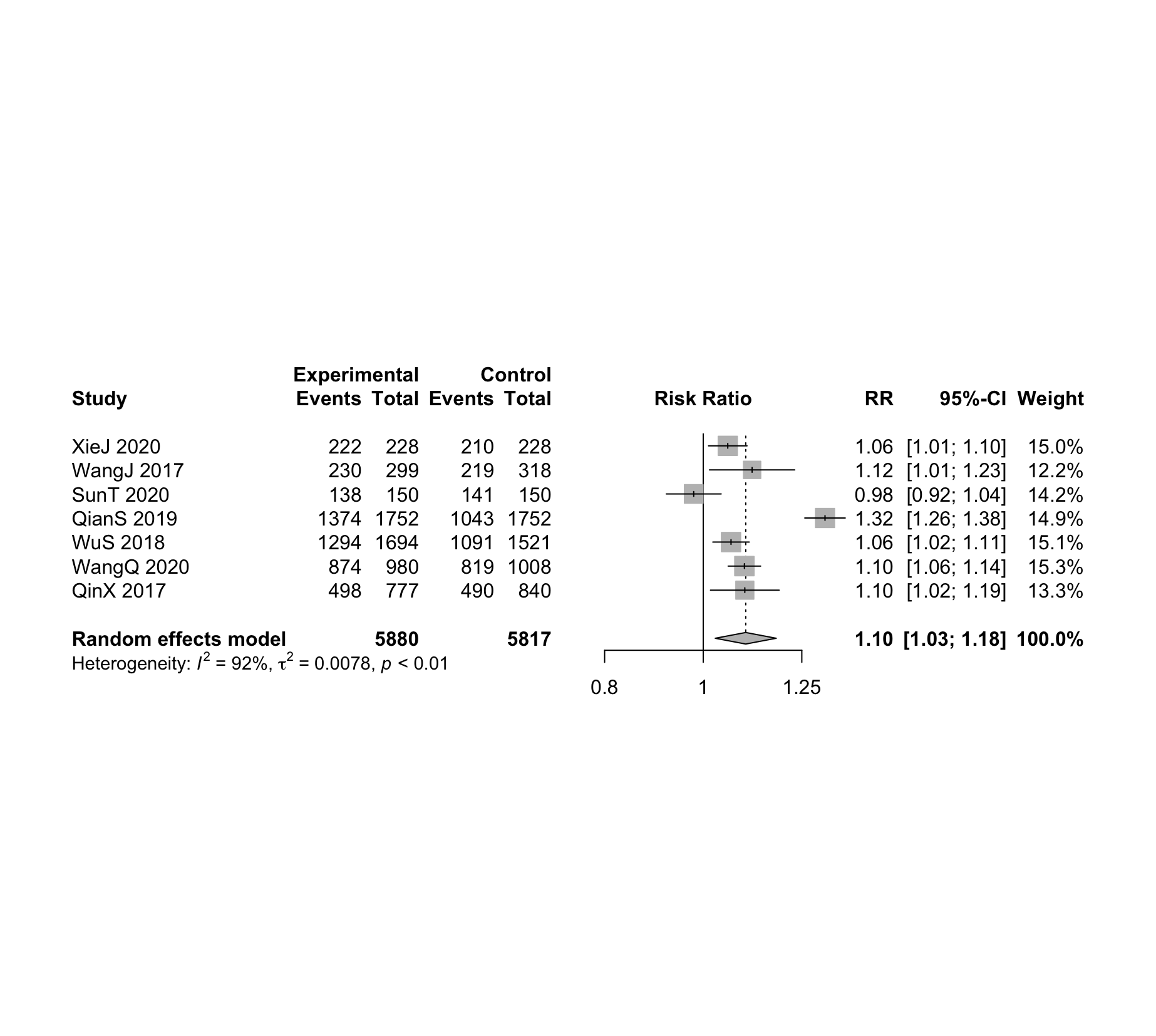

Supplement: S17 Fig — (PNG) [file pone.0304221.s021.PNG]

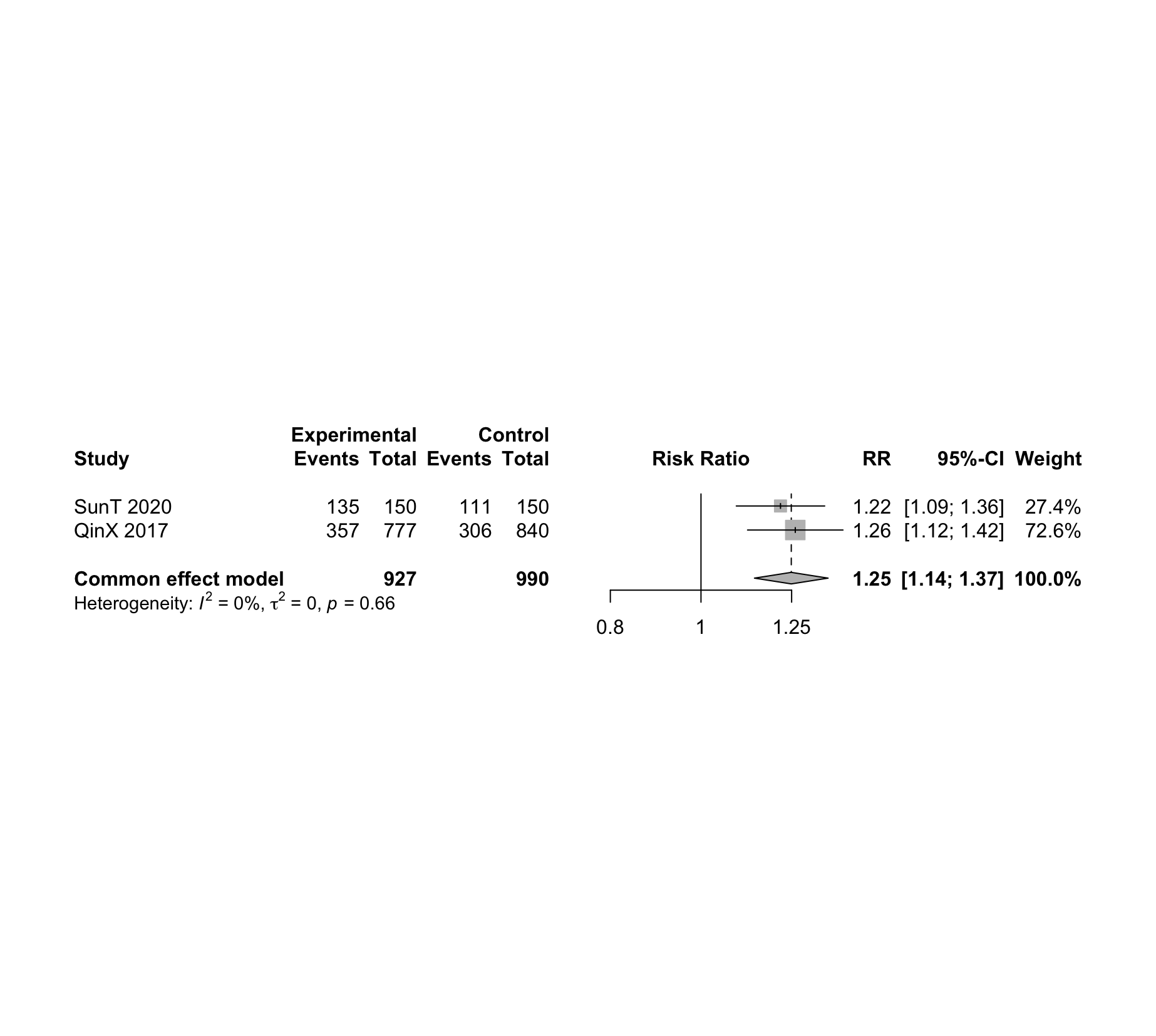

Supplement: S18 Fig — (PNG) [file pone.0304221.s022.PNG]
